# Structure and hydrodynamics of a DNA G-quadruplex with a cytosine bulge

**DOI:** 10.1093/nar/gky307

**Published:** 2018-05-01

**Authors:** Markus Meier, Aniel Moya-Torres, Natalie J Krahn, Matthew D McDougall, George L Orriss, Ewan K S McRae, Evan P Booy, Kevin McEleney, Trushar R Patel, Sean A McKenna, Jörg Stetefeld

**Affiliations:** 1 Department of Chemistry, University of Manitoba, Winnipeg, Manitoba R3T 2N2, Canada; 2 Alberta RNA Research and Training Institute, Department of Chemistry and Biochemistry, University of Lethbridge, Lethbridge, Alberta T1K 3M4, Canada; 3 DiscoveryLab, Medical Sciences Building, University of Alberta, Edmonton, Alberta T6G 2H7, Canada; 4 Department of Microbiology, Immunology and Infectious Diseases, Cumming School of Medicine, University of Calgary, Calgary T2N 1N4, Alberta, Canada; 5 Department of Biochemistry and Medical Genetics, University of Manitoba, Winnipeg, Manitoba R3T 2N2, Canada

## Abstract

The identification of four-stranded G-quadruplexes (G4s) has highlighted the fact that DNA has additional spatial organisations at its disposal other than double-stranded helices. Recently, it became clear that the formation of G4s is not limited to the traditional G_3+_N_L1_G_3+_N_L2_G_3+_N_L3_G_3+_ sequence motif. Instead, the G_3_ triplets can be interrupted by deoxythymidylate (DNA) or uridylate (RNA) where the base forms a bulge that loops out from the G-quadruplex core. Here, we report the first high-resolution X-ray structure of a unique unimolecular DNA G4 with a cytosine bulge. The G4 forms a dimer that is stacked via its 5′-tetrads. Analytical ultracentrifugation, static light scattering and small angle X-ray scattering confirmed that the G4 adapts a predominantly dimeric structure in solution. We provide a comprehensive comparison of previously published G4 structures containing bulges and report a special γ torsion angle range preferentially populated by the G4 core guanylates adjacent to bulges. Since the penalty for introducing bulges appears to be negligible, it should be possible to functionalize G4s by introducing artificial or modified nucleotides at such positions. The presence of the bulge alters the surface of the DNA, providing an opportunity to develop drugs that can specifically target individual G4s.

## INTRODUCTION

Nucleic acid G-quadruplexes (G4s) adopt a four-stranded structure where four guanines from different G-tracts form a planar tetrad strengthened by hydrogen bonds between Watson–Crick and Hoogsteen faces of adjacent guanines (1). This unique arrangement enables highly efficient base stacking between multiple successive guanine tetrads and represents the major stabilizing feature of G4s. Further reinforcement is provided by a requisite monovalent or divalent cation (typically K^+^) that occupies the central channel between stacked tetrads, reducing electrostatic repulsion from the O_6_ oxygens of the guanines (1). The consecutive G-tracts of three or more guanylates can be located either on a single nucleic acid molecule (unimolecular G4) or be distributed on two (bimolecular G4) or four (tetramolecular G4) separate macromolecules. The G-tracts can adopt a parallel (where all strands run in the same direction), antiparallel (where strands have alternate directionality), or hybrid (where three strands run in one direction and the fourth the opposite) orientation. In the case of unimolecular or bimolecular G4s, the G-tracts are joined by loops ranging from a single nucleotide to several hundred nucleotides. Strand orientation significantly impacts loop connectivity, as parallel G4s require loops to connect the top tetrad to the bottom, whereas in antiparallel orientation the loops connect strands on the same tetrad. The G-tracts themselves can also be interrupted by nucleotides, forming a bulge, further increasing the structural heterogeneity of different G4s (1,2). We counted (as of October 2017) 231 entries in the PDB (3) for high-resolution structures of G4 forming nucleic acids; of these 90 were determined by X-ray crystallography. This catalogue contains many examples of different strand orientations and loop composition, but only six examples of G4s with bulged nucleotides. There exist many excellent reviews on G4 structures and their biophysical properties (1,4–6). A recent biophysical study focused solely on the effect of bulges on G4s in a systematic manner (2).

The major sources of structural heterogeneity amongst the population of DNA G4 species are created by differences in loop sequence, length, orientation, and bulged nucleotides within the G-tracts themselves. Progress towards the discovery of small molecules that target specific DNA G4 species might be expedited through a better understanding of these structural features of G4. The major obstacle facing the discovery of such ligands is their specificity; an effective G4 ligand must demonstrate high selectivity for a specific G4. A prerequisite for the rational design of such ligands is the structural characterization of a wide variety of G4 structures. Furthermore, a better understanding of how DNA G4 structures accommodate loops and bulges could see their incorporation in nanoscale material for drug delivery (7) and single molecule probes for biophysical assays (8,9) or medical diagnostics (10) among other applications.

Both DNA and RNA G4s have been associated with a number of important biological functions and there exist multiple review articles detailing the relevance of G4 as regulatory elements (11–13). In this work, we study the DNA version of an RNA G4 that forms in the 5′-end of the human telomerase RNA component (hTR). Formation of quadruplex in this region interferes with P1 helix formation, which is vital for an active telomerase enzyme (14). An RNA G4 resolving enzyme (DHX36) can interact with the 5′-region of hTR and unwind the G4, promoting P1 helix formation, an activity that is associated with increased telomerase activity (15–17). We have previously demonstrated that both the RNA and DNA versions of the quadruplex formed in the first 20 bases of hTR are of parallel topology and have a highly similar bun-shaped structure, as determined by SAXS (18). Furthermore, NMR chemical shift perturbation experiments with a small G4-interacting portion of DHX36 indicate that both RNA and DNA quadruplexes interact with the same amino acids of DHX36 (18).

Here we used the DNA version of hTR 1–20 in complex with the DHX36 G4-interacting peptide in crystallization trials. Although we were unable to observe any electron density from the peptide, the crystals grown showed diffraction from nucleic acid component to sufficient resolution for its high-resolution structure to be determined. This G4 contains a cytosine bulge in one G-tract and we assess its impact on the G4 structure. We further support the rigour of our structural data using an integrated biophysical approach to characterize the G4 using size exclusion chromatography (SEC), sedimentation velocity (SV), SEC coupled multi-angle light scattering (SEC-MALS), dynamic light scattering (DLS) and SEC coupled small angle X-ray scattering (SEC-SAXS) at multiple concentrations to show that it exists predominantly as a dimer across a large concentration range. Finally, we compare the structural features of the hTR 1–20 DNA G4 to other bulged G4 structures and present a thorough analysis of backbone torsion angles, identifying a unique range of γ torsion angles amongst bulged G4s. The easily accommodated nucleotide bulge presents a unique site to add functional groups or to facilitate specific recognition by novel G4 ligands.

## MATERIALS AND METHODS

### Synthesis and purification

Nucleotides 1-20 of human telomerase RNA component with sequence d(GGG TT G**C**GG A GGG T GGG CCT) were chemically synthesized as DNA and PAGE purified by AlphaDNA, Montreal, Canada in 15 μmol scale. We refer to it as *hTR 1-20 DNA* in this publication. G4s were formed by suspending the DNA at a concentration of 5 μM in 20 mM HEPES, pH 7.5, 100 mM KCl, heating the sample to 95°C for 10 min, followed by slow cooling in the water bath. The G4s were then purified on a HiLoad Superdex 75 26/60 size exclusion chromatography (SEC) column, yielding two distinct species, c1 and c2 (Supplementary Figure S1A). The sharp c2 elution peak contained a single, crystallizable conformation of G4. The broad c1 elution peak showed features of G4 also, but likely included multiple conformations and did not crystallize. More c2 could be obtained by re-heating the c1 fraction and repeating the purification. However, the yield of c2 diminished with each cycle (Supplementary Figure S1B).

### Sample preparation for biophysical studies

Purified hTR 1-20 DNA c2 in 20 mM HEPES, pH 7.5, 100 mM KCl was concentrated to 13 mg/ml using an Amicon^®^ concentrator with 3000 Da molecular weight cut-off and then diluted to the desired final concentrations with the same buffer. This buffer was used for all biophysical analysis except when indicated otherwise.

### Spectropolarimetry

All circular dichroism (CD) spectra were recorded as previously described in detail (18). Briefly, data were collected on a calibrated J-810 spectropolarimeter (Jasco Inc., Easton, MD, USA) in a 1.0 mm (220–200 nm) or 0.1 mm (220–180 nm) quartz cell at a concentration of 30–50 μM (1.0 mm cell) or 200 μM (0.1 mm cell) in 20 mM sodium phosphate, pH 7.5, 100 mM KF. The spectra were measured in triplicate, averaged, buffer subtracted and normalized by the number of nucleotides per unit volume (Supplementary Figure S2).

### Dynamic light scattering

To assess sample homogeneity, dynamic light scattering (DLS) data were collected on a Zetasizer Nano-S instrument (Malvern Instruments Canada, Montreal, QC, Canada), equipped with a 633 nm (red) He-Ne Laser and a 173° backscatter detector (19) from the same hTR 1-20 DNA samples that were used for sedimentation velocity experiments (Supplementary Figure S3). The samples were centrifuged at 13000 rpm for 5 min in an Eppendorf™ MiniSpin™ centrifuge and then filtered through a 0.1 μm Millipore Ultrafree^®^-MC filter immediately before transfer to the 3 × 3 mm quartz cell (Hellma Canada Ltd., Markham, ON, Canada). The temperature was equilibrated to 20°C for 5 min before starting the measurements. The small size of the G4 required very long measurement times and only sample concentrations above 1.0 mg/ml concentrations provided enough signal. The sample preparations were highly homogenous and contained only trace amounts of aggregates or higher order oligomers.

### Static light scattering

To determine the molecular mass, we used an in-line Dawn^®^ Heleos^®^ II multi-angle static light scattering (MALS) detector (Wyatt Technology, Santa Barbara, CA, USA) in conjunction with a 24 ml Superose 12 10/300 GL SEC column driven by an ÄKTA pure FPLC system (GE Healthcare, Toronto, ON, Canada). Sample concentration was monitored by a 2 mm multi-wavelength UV flow cell (GE Healthcare) and an in-line Optilab T-rEX differential refractometer (Wyatt Technology). 200 μl sample was injected into the buffer equilibrated column at a concentration of 7.66 mg/ml using a flow rate of 0.3 ml/min. The UV signal was recorded at a wavelength of 290 nm, where the hTR 1-20 DNA c2 G4 has an absorption coefficient of 58756 M^-1^ cm^-1^ (Supplementary Figure S5A). The Superose 12 column has a separation range of 1–300 kDa.

### Sedimentation velocity

Sedimentation velocity (SV) profiles were measured on a ProteomeLab™ XL-I analytical ultracentrifuge (Beckman Coulter Canada, Mississauga, ON, Canada) using an An-50 Ti 8-place rotor, a rotor speed of 42 000 rpm, a temperature *T* of 20°C and sample concentrations of 11.12, 8.90, 4.45, 2.00, 1.00, 0.50, 0.10, 0.05, 0.01 and 0.005 mg/ml, corresponding to a range of 1.8 mM to 800 nM. Samples were dialysed in the reference buffer overnight and then diluted to the desired concentration. 400 μl of sample and buffer was loaded into the respective channel of the double-sector centrepiece. The 8-place An-50 Ti rotor with samples was allowed to equilibrate to the pre-set temperature for at least 2 h. Radial scans were collected every 10 min for 24 h. Two-dimensional distributions (20) *c*(*s, f_r_*) of sedimentation coefficient *s* and frictional ratio *f_r_* were calculated in *SEDFIT* using a grid with 20 evenly spaced points along *f_r_* and 50 variably spaced points along *s*. Between 0.5 and 4.0 S, the points were evenly spaced with a distance of 0.1 S and from there increasingly further apart up to 20 S. To overcome the 2 GiB addressable memory limit of *SEDFIT*, a 32-bit program, we removed all points of the radial scans outside the usable data range prior to loading them into *SEDFIT*, but we kept the full radial resolution. We also removed every uneven scan after 18 h (scan # 108), increasing the scan time interval from 10 to 20 min. The obtained *c*(*s, f_r_*) distributions established the presence of a major and a minor species within a 4.45–0.005 mg/ml concentration range (Supplementary Figure S8A–M, Supplementary Table S5A–M, Figure 4A). The data of the two highest sample concentrations (11.12 and 8.90 mg/ml) were excluded from further analysis as explained in the Results section. We then calculated the apparent *s*, apparent molecular mass *M*, molecular fractions and measurement uncertainties of both species at each sample concentration by fitting these parameters to the combined Lamm and Svedberg equations using the *Species Analysis with Mass Conservation Restraints* model implemented in *SEDPHAT* (21,22) (Figure 4B and C). The obtained values were then linearly extrapolated to infinite dilution and converted to standard conditions (pure water at 20°C) using Equation (1) with a buffer density *ρ_T,b_* of 1.0045 g/cm³, a buffer viscosity *η_T,b_* of 0.0101543 P, and for the G4 a previously reported (23) (determined from a 22-nucleotides G4 in 10 mM Tris, pH 8.0, 1 mM EDTA, 75 mM KCl) partial specific volume ${\bar{\nu }_{T,b}}$ = ${\bar{\nu }_{20^\circ C,w}}$= 0.541 ± 0.019 cm^3^/g to obtain *s*^0^_20°C_*_,w_* and *M^0^* (Table 1).

(1) (Ref. 24) \begin{equation*}{s_{20^\circ {\rm C},w}} = {s_{T,b}}\ \frac{{{\eta _{T,b}}}}{{\ {\eta _{20^\circ {\rm C},w}}}}\ \frac{{\left( {1\ - \ {{\bar{\nu }}_{20^\circ {\rm C},w}}\ {\rho _{20^\circ {\rm C},w}}} \right)}}{{\left( {1\ - \ {{\bar{\nu }}_{T,b}}\ {\rho _{T,b}}} \right)}}\nonumber \\ \end{equation*}

**Table 1.** Hydrodynamic and physical properties

Property	Monomer	Dimer	Method
*Experimental*			
Radius of gyration (*R_g_*), nm		1.419 ± 0.004	In-line SEC-SAXS (*P(r)* distribution)
Largest dimension (*D_max_*), nm		4.619	In-line SEC-SAXS (*P(r)* distribution)
Extrapolated intensity (*I(0)*) at scattering angle 0		0.05469 ± 0.00006	In-line SEC-SAXS (*P(r)* distribution)
Extrapolated hydrodynamic radius (*R_h_*^0^_20°C_), nm	1.2 ± 0.2	2.0 ± 0.1	SV, species analysis
Extrapolated sedimentation coefficient (*s*^0^_20°C_*_,w_*), S	1.5 ± 0.2	2.70 ± 0.02	SV, species analysis
Extrapolated sedimentation coefficient (*s*^0^_20°C_*_,w_*), S	1.5 ± 0.2	2.69 ± 0.03	SV, *c*(*s, f_r_*) analysis
Extrapolated sedimentation coefficient (*s*^0^_20°C_*_,w_*), S	1.4 ± 0.2	2.71 ± 0.04	SV, *c*(*s*) analysis
Extrapolated molecular mass (*M*^0^), kDa	4.7 ± 0.9	13.5 ± 0.7	SV, species analysis
Extrapolated molecular mass (*M*^0^), kDa	5.8 ± 0.6	15.0 ± 0.9	SV, *c*(*s, f_r_*) analysis
Molecular mass (*M*), kDa		14 ± 1	In-line SEC-MALS
Molecular mass (*M*), kDa		14	In-line SEC-SAXS
*In-line SEC-SAXS low resolution structures* ^a^			
Radius of gyration (*R_g_*), nm		1.4219 ± 0.0005	*DAMMIN*
Largest dimension (*D_max_*), nm		4.83 ± 0.06	*DAMMIN*
Volume (*V*), nm^3^		19.1 ± 0.2	*DAMMIN*
Hydrodynamic radius (*R_h_*_20°C_*_,w_*), nm		1.805 ± 0.008	*HYDROPRO*
Sedimentation coefficient (*s*_20°C_*_,w_*), S		2.86 ± 0.02	*HYDROPRO*
*X-ray crystal structure*			
Radius of gyration (*R_g_*), nm	1.205	1.423	*HYDROPRO*
Largest dimension (*D_max_*), nm	4.37	4.79	*HYDROPRO*
Volume (*V*), nm^3^	8.82	17.22	*HYDROPRO*
Hydrodynamic radius (*R_h_*_20°C_*_,w_*), nm	1.54	1.94	*HYDROPRO*
Sedimentation coefficient (*s*_20°C_*_,w_*), S	1.67	2.67	*HYDROPRO*
Molecular mass (*M*), kDa	6.3643 (G4_1_ K^+^_2_)	12.7677 (G4_2_ K^+^_5_)	Atomic composition

The uncertainties are expanded and represent 95% confidence intervals.

^a^Mean values of the nine final *DAMMIN* structures.

Graphical representations of the residuals and fits to the sedimentation velocity scans as well as the *c*(*s, f_r_*) and *c*(*s, M*) distributions were generated with *GUSSI* (25) and can be found in Supplementary Figure S8A-M. The HEPES buffer parameters were calculated with the software package *SEDNTERP 2* (26,27).

### Small angle X-ray scattering

In-house small angle X-ray scattering (SAXS) data were acquired and processed as previously described in detail (28). Briefly, data were collected in batch mode (capillary) on a Rigaku 3-pin hole camera (S-MAX3000) equipped with a Rigaku MicroMax+002 microfocus sealed tube (Cu-K_α_ radiation at 1.54 Å) and a Confocal Max-Flux (CMF) optics operating at 40 W, using a calibrated 200-mm multi-wire 2D detector. Over the past few years, we collected full datasets (several concentrations) of G4s from three separate hTR 1-20 DNA synthesis batches using X-ray exposure times of 2–4 h. Data from the same synthesis batch were reduced with *SAXSGUI* (JJ X-Ray Systems ApS, Hoersholm, Denmark), averaged and buffer subtracted with *PRIMUS* (29). The datasets comprised the following concentrations: 4.8, 3.6 and 3.0 mg/ml (dataset published in Meier *et al.* (18)); 4.8, 3.3, 2.6 and 1.7 mg/ml (dataset from 2012); 2.5, 3.5 and (another) 3.5 mg/ml (dataset from 2016).

Synchrotron SEC-SAXS data were collected at the B21 beamline at the Diamond Light Source (Didcot, UK) using an in-line Agilent 1200 (Agilent Technologies, Stockport, UK) HPLC system connected to a specialized flow cell. 50 μl hTR 1-20 DNA c2 G4 with a concentration of 5.58 mg/ml was injected into a buffer equilibrated 4.6 ml Shodex KW402.5-4F size exclusion column using a flow rate of 0.16 ml/min. The column diluted the sample ∼3-fold (∼2 mg/ml) before it arrived at the flow cell. Each frame was exposed for 3 s. Nine frames of the sample peak region were integrated, buffer subtracted and merged using the ScÅtter software package (30).

*Ab initio* low-resolution structures were calculated with software from the *ATSAS* suite (31). We first generated sets of 20 models with *DAMMIF* (32), using identical parameters within each set but a different random seed for each model. No symmetry was enforced (P1). Between the sets we slightly varied the maximum distance *D_max_* (4.400, 4.614 and 4.619 nm in case of the SEC-SAXS data) and the number of data points when calculating the pair distance distribution *P*(*r*) in *GNOM* (33) and we tried the three shape classes *compact-hollow, compact* and *unknown* implemented in *DAMMIF*. The models in each set were then aligned, averaged and filtered using *SUPCOMB* (34) and *DAMAVER* (35) which provided an individual fixed core for each final structure calculated in *DAMMIN* (36). From the nine final SEC-SAXS structures, we uploaded those three based on the *P*(*r*) distribution with the highest quality estimate (*Dmax* = 4.619 nm) to the *Small Angle Scattering Biological Data Bank* SASBDB (37) where they are accessible under ID SASDCC8.

The molecular mass in Table 1 was determined from the merged scattering data using SAXSMoW2 (38) and the result was corrected for the appropriate mass density of the G-quadruplex DNA (1.85 g/cm³ = ${\raise0.7ex\hbox{$1$} \!\mathord{/ {\vphantom {1 {\bar{\nu }}}}} \!\lower0.7ex\hbox{${\bar{\nu }}$}}$, see SV analysis).

### Calculation of hydrodynamic parameters

To verify our low resolution SAXS structures, we calculated the hydrodynamic parameters (hydrodynamic radius *R_h_*, sedimentation coefficient *s*) from the bead models using the program *HYDROPRO* (version 10) (39–41). An important parameter for *HYDROPRO* is the *atomic element radius* (*AER*), i.e. the radius of the beads in the SAXS (primary) model. This parameter can be calculated from the ‘*dummy atom volume’ V_DA_* reported in the header of each PDB file produced by *DAMMIF* by using Equation (2). For *DAMMIN* models, *V_DA_* is reported as ‘*average volume per atom*’. The radius of gyration *R_g_*, largest dimension *D_max_*, excluded volume *V_DAM_* and estimated molecular mass *M* can also be found in the header.

(2) \begin{equation*}AER = \sqrt[3]{{\frac{{3\ {V_{DA}}}}{{4\ \pi }}}}\end{equation*}

All our *DAMMIF* models had a *V_DA_* of 5.661 Å^3^ that yielded an *AER* of 1.106 Å, whereas the *DAMMIN* models contained a *V_DA_* of 11.056 Å^3^ that corresponded to an *AER* of 1.382 Å. Using these values, *HYDROPRO* produced particle volumes that matched very closely to the volumes reported by the SAXS models and also reproduced *R_g_* and *D_max_* correctly. More information about the *AER* parameter determination can be found in the supplementary information. For the X-ray crystal structure, we used an *AER* parameter of 2.54 Å, a value that was calibrated (42) with several G4 structures from the protein database (3) and experimental data. For all cases, we used the atomic-level primary model calculation (INDMODE = 1) with eight shells, where the innermost shell contained 200–300 and the outermost shell 1800–3000 minibeads.

### Crystallization and structure solution

Crystals were formed with an initial complex of 5 mg/ml hTR 1-20 DNA c2 and a 1:1 molar ratio of human DHX36(53-105) peptide (residues 53-105 of ATP-dependent RNA helicase DHX36, UniProtKB (43) Q9H2U1 or DHX36_HUMAN) by hanging drop vapour diffusion with drop sizes of 2+2 μl in 2.5 M NaCl, 10% (v/v) ethanol, 0.1 M sodium cacodylate, pH 6.5 at 20°C. Crystals emerged after 3 months and grew as fragile stacks of plates. Before data collection, crystals were soaked in reservoir solution containing 15% ethylene glycol and 5 mM of both HgCl_2_ and PtCl_4_ for 24 h. This procedure separated the stacks into fewer layers of plates that could be picked up for cryogenic cooling to 100 K in a Rigaku X-stream™ 2000 cold stream. X-ray diffraction images of the crystals were collected in 1° wedges with 10-min exposure on a Rigaku MicroMax™-007 HF equipped with an R-AXIS IV++ detector and a Rigaku Osmic™ Confocal Max-Flux^®^ multilayer mirror using a Cu Kα source. Due to the presence of two crystals plates in the loop during data collection, two non-overlapping lattices were present. The lattices were processed individually, then scaled and merged together using the *HKL-2000* package (44). The space group was determined with *Pointless* (45,46). Phases were determined by molecular replacement in *Phaser* (47) using the core of another parallel G4 structure (PDBID 1XAV (48)) as a search model. *Coot* (49) was used to build the atomic model into the electron density map, followed by refinement with *Refmac5* (50). We wrote scripts to perform data conversion and to generate data statistics by taking advantage of the *Computational Crystallography Toolbox* (*CCTBX*) (51) and we also used software of the *Collaborative Computational Project Number 4* (*CCP4*) (52). The crystals only contained DNA; no traces of the peptide could be found. We therefore monitored the stability of the DHX36(53–105) peptide by SDS PAGE and determined that fragmentation started to occur after 5 days at room temperature and was essentially complete after only one month. The fully refined coordinates and the structure factors were deposited in the protein database with PDBID 5UA3.

### Nucleic acid geometry and visualization

We used the program *DSSR* (53) of the *3DNA* suite (54) to analyse the nucleic acid backbone and the base pair geometry from the 3D structures. We reported the ‘simple’ base-pair parameters for buckle, propeller twist and stagger which are more intuitive for non-canonical base-pairs than the classic base-pair parameters as explained in the program manual and the 3DNA website (http://x3dna.org/highlights/details-on-the-simple-base-pair-parameters, http://x3dna.org/articles/simple-parameters-for-non-Watson–Crick-base-pairs). We wrote an R (55) script that automatically creates a backbone angle plot from the output of the *DSSR* program. The script can be downloaded from the *3DNA* forum at http://x3dna.org. The nucleic acid was visualized in *PyMOL* and the *dssr_block* plugin (The PyMOL Molecular Graphics System, Version 2.0, Schrödinger, LLC, https://pymol.org/). All other figures in this publication were created in *QtiPlot* (QtiPlot—Data Analysis and Scientific Visualization, http://www.qtiplot.com).

## RESULTS AND DISCUSSION

Based on a well-characterized short 20-nt region from the 5′-end of the human telomerase RNA (hTR 1-20), we chose to study the DNA equivalent from a biophysical perspective. Purification of this chemically synthesized DNA by size exclusion chromatography resulted in two species with similar but distinct sizes; referred to as c1 (eluting first, ∼65%) and c2 (eluting second, ∼35%), see Supplementary Figure S1.

### Spectropolarimetry suggests a pure parallel strand orientation for c2

We have previously reported that both, c1 and c2 exhibit circular dichroism (CD) spectra with the general features of typical parallel G4s (56), characterised by a dominant positive peak at 263 nm and a negative peak at 242 nm (18) (see Supplementary Figure S2). However, c1 and c2 have distinguishable spectral features, suggesting differences in overall structure. The tailing shoulder near 290 nm of the c1 species is likely caused by a mixture of syn- and anti-glycosidic bond dihedrals, indicative for the presence of some antiparallel conformations, whereas the c2 species has a much sharper peak at 263 nm indicative of a uniform parallel strand orientation with only anti-glycosidic bonds. Thermal melting of the G4s resulted in a gradual disappearance of these spectral features (characteristic of guanine unstacking) with c1 demonstrating an elevated estimated midpoint (70°C) relative to c2 (64°C) (18). Both midpoint values were significantly higher than that expected for single- or double-stranded nucleic acid structures and are consistent with G4s.

### High-resolution structure of the hTR 1-20 DNA

**Figure 1.**
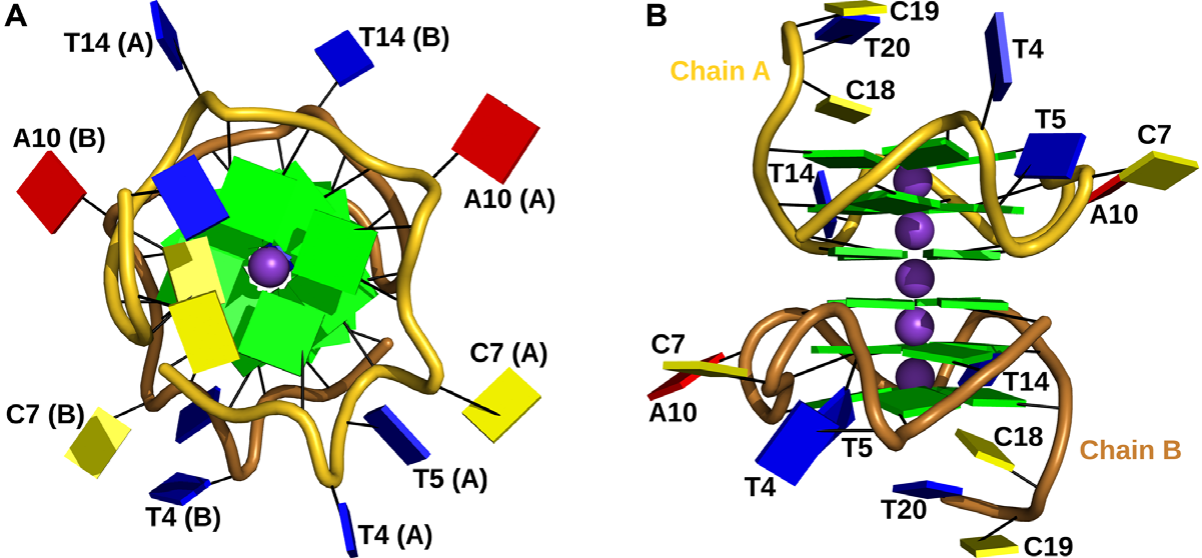
Cartoon representation of the hTR 1-20 DNA c2 X-ray crystal structure with the backbone of chain A coloured in gold and the one of chain B in copper. The bases are represented as cartoon blocks, cytosines in yellow, guanines in green, thymines in blue and adenines in red. The bases of the loops, bulges and tails are labelled. The potassium ions are shown as magenta spheres. (**A**) View on top of the CCT tail and 3′-tetrad face of the G4. (**B**) Side view of the guanylate core with the bulges, loops and tails facing out.

**Figure 2.**
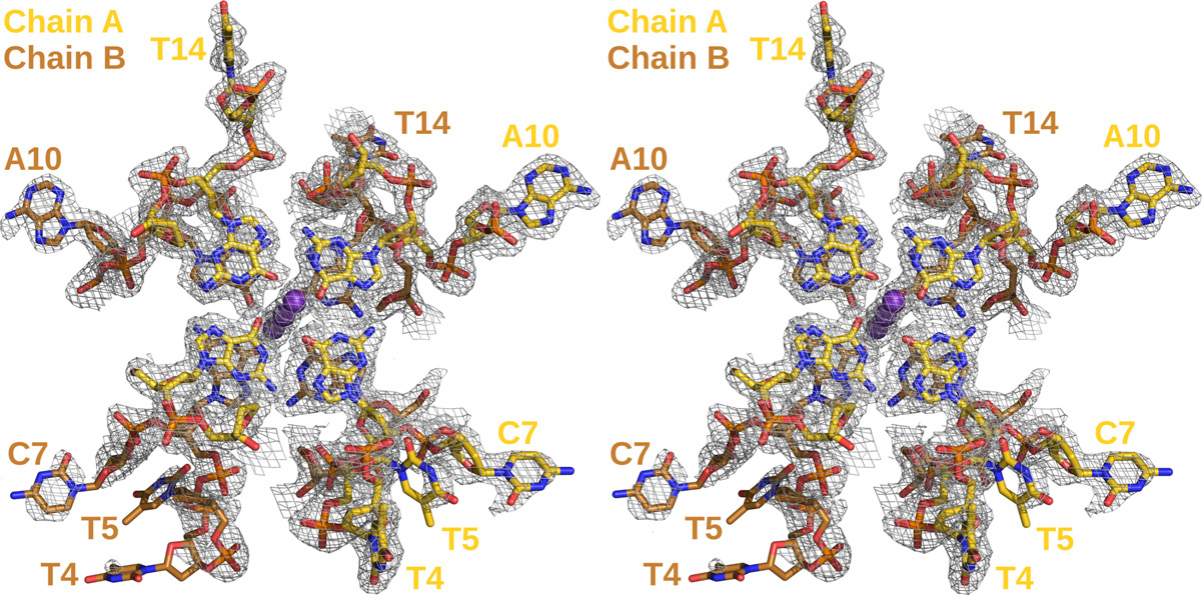
Stereo image of a fraction of the high-resolution X-ray crystal structure of the hTR 1-20 DNA c2 dimer built into the electron density map. Only the 5′-tetrads with their potassium ions as well as the loops and bulges are displayed. The electron density is shown at a root-mean-square deviation of 1.0 σ (0.31 e/Å^3^). The chain backbone A is coloured in gold, chain B in copper, potassium ions are represented as magenta spheres. The bases of loops and bulges are labelled.

Of the two species c1 and c2, only the latter could be crystallized. This is not surprising, since c1 represents likely a heterogeneous mixture of parallel and antiparallel G4s. The crystal structure of c2 has a traditional resolution of limit 1.88 Å (shell where *I/σ*(*I*) = 2.0) with a single dimer occupying the asymmetric unit (Figure 1, Supplementary Table S1). The G4 crystallized in space group *P*1. Consistent with spectropolarimetry results, each G4 adopts a parallel orientation with all glycosidic bond angles (*χ*) adopting the anti-conformation, which results in a head-to-tail stacking of the three guanine tetrads. The centre of each of the five tetrad stacks that comprise the dimer is occupied by a potassium ion, forming a linear grid with a distance of 3.3–3.5 Å between the atoms. Each tetrad within an individual G4 is offset by 30° counter-clockwise to the previous one (looking from head to tail down the axis formed by the central potassium ions, Figure 1A), resulting in a spiral orientation of the guanine bases. The two G4s that comprise the dimer stack in a head-to-head orientation via the guanine tetrads formed by the 5′-nucleotides (Figures 1B and 2).

A tail of three nucleotides protrudes out at both 3′ ends of the dimer (Figure 1B). This tail is comprised of nucleotides 18–20 with the sequence d(CCT) that follow the last G-tract. Nucleotides 19 and 20 of chain B have multiple conformations, but the electron density was only clear enough to build one of them (occupancy 0.8). The equivalent nucleotides in chain A are well defined and their bases and the backbone stack on top of the 3′-tetrad of chain B (contacts to dG3, dG9 and dG13) of the nearest symmetry related dimer. dC18 of both chains stacks on top of the 3′-tetrad of its own respective chain (contact to dG17). The base of dC19 (sym. chain B) stacks on the centre of the 3′-tetrad (chain A) while its neighbour, dT20 (sym. chain B), intercalates between the bases of dC18 (chain A) and dT20 (chain A) and is further stabilized by a hydrogen bond to the phosphate of dT20 (chain A). A network of hydrogen bonds stabilizes the triangle formed by dC18 (sym. chain B), dC19 (chain A) and dC20 (chain A) and triplet of hydrogen bonds connects the bases of dC18 (chain A) and dC19 (sym. chain B). This tight interaction network between the tails impedes access to the 3′-tetrad faces.

In contrast, the 5′-end of the hTR 1-20 starts directly with the first guanine of G-tract 1 and consequently, there are no nucleotides to block access to the 5′-tetrad face, making it available for tetrad stacking. The 5′-tetrad faces are almost planar, whereas the base pairs forming the 3′-tetrads are buckled, forming a concave (convex, if viewed 5′-3′) depression towards the potassium ion (Figure 1B). The latter provides a less ideal surface for the base stacking of a planar tetrad. Limited access to the 3′-tetrad, its concave surface and free access to the planar 5′-tetrad all favour 5′-5′ stacking of the G4 dimer. Searching the PDB data bank (3), we found several other examples of parallel DNA G4 with 5′-5′ tetrad stacking (e.g. PDBIDs 352D (57), 3CCO (58), 2LE6 (59), 3QSF (60), 3QSC (60)) and at least one example with a 3′-3′ stacking (PDBID 4U92 (61), Figure 6D), confirming that both stacking modes are possible.

Buckled tetrads were already observed in the early structures deposited in the PDB (3), e.g. the 5′-5′ stacked tetramolecular parallel G4 [d(TGGGGT)]_4_ with PDBID 352D (57) and are a recurrent theme. Among 5′-5′ stacked parallel G4s, the tetrads at the stacking interface can be both planar (our structure), one tetrad planar and one convex (PDBID 352D (57)) or both tetrads saddle shaped (3CCO (58), 3QSF (60)). Deviation from planarity can also be caused by propeller twists (2LE6 (59)) or staggers. We define tetrads as planar if the buckles and propeller twists are less than 5°. Convex bowl-shaped tetrads form if all buckles are positive. Saddle-shaped tetrads are obtained if they contain alternating positive and negative buckles. We have calculated these base pair parameters for our and the aforementioned structures (see Supplementary Table S3). Buckled tetrads are not unique to stacked G4 pairs, but also occur in unimolecular monomeric G4s (e.g. PDBID 2LEE (62)). Compared to other unimolecular G4s in the PDB, the tetrad geometries in our structure are remarkably symmetric, even though the presence of the asymmetric loops and the bulge would suggest otherwise. Even the above mentioned simple symmetric sequence [d(TGGGGT)]_4_ has an asymmetric structure (PDBID 352D (57)).

Our G4 core is built from the four G-tracts in the DNA sequence: tract 1 (dG1–dG3), tract 2 (dG6, dG8, dG9), tract 3 (dG11–dG13), and tract 4 (dG15–dG17). Unlike tracts 1, 3, and 4 that employ continuous guanines, tract 2 is interrupted by dC7, which adopts a looped-out type I bulge conformation in both chains (Figure 2). Electron density around the bases of the cytosine bulges is ill defined and the atoms of the bases have high B-factors, indicating conformational freedom. Rotation of the base around the glycosidic bond is partly restricted, however, through crystal contacts. The base planes of dC7 (chain A), dC7 (chain B) and dT4 (chain B), each originating from different symmetry molecules, can be oriented such that they form a triple π-π stack. This arrangement is indeed partially occupied (∼0.6) and visible in the electron density. Whether the cytosine bases (dC7) occupy the syn- or anti-conformation in this arrangement is unclear. Most of the G4 loops show strong electron density. However, having no interaction partners, dT4 in chain B is largely disordered (deoxyribose and base) with only the backbone phosphates visible. Interestingly, dT4 in chain A has very defined electron density, packing snugly onto dT14 of a symmetry mate. The base of dT5 in both chains seems to be rotating around its glycosidic bond.

**Figure 3.**
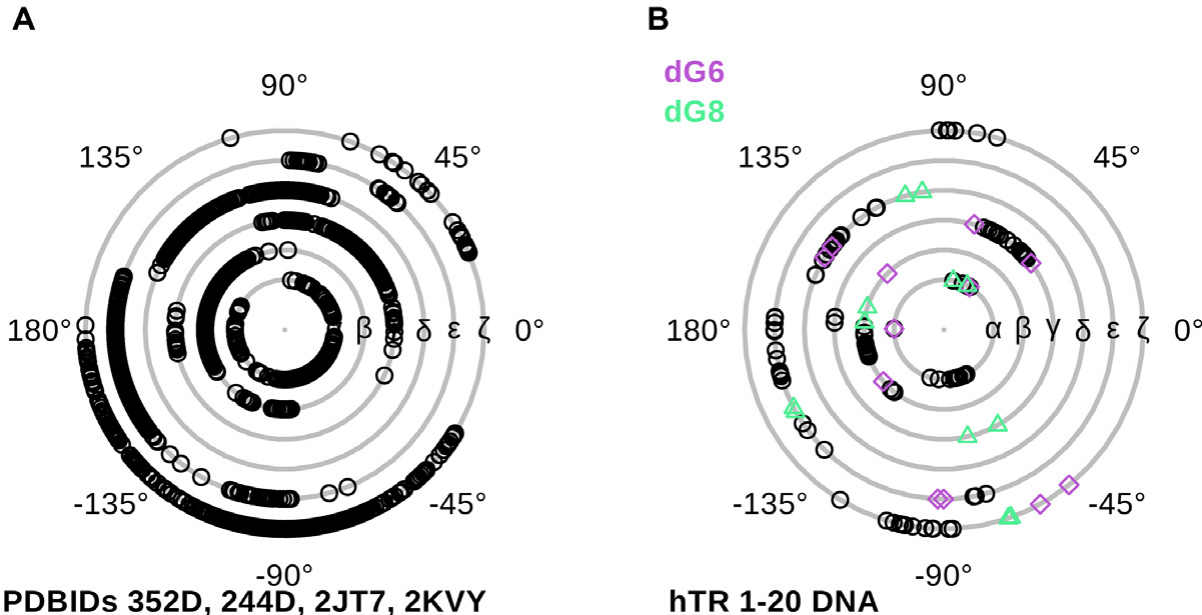
(**A**) Backbone torsion angle plot of the ideal G4 sequence [d(TGGGG)]_4_ without loops or bulges. Only the core guanylates are shown. The two X-ray structures 244D (63) and 352D (57) contain four G4s each, providing a total of 128 values for each torsion angles of every guanylate. PDBIDs 2JT7 (64) and 2KVY (65) contain 10 NMR models each and provide another total of 320 values. (**B**) Backbone torsion angle plot of the guanylates forming the G4 core of hTR 1-20 DNA. dG6 that precedes the cytosine bulge (dC7) is coloured in orchid and dG8 that follows the bulge is in seagreen.

To assess how the cytosine bulge affects the G4 geometry, we compared the backbone torsion angles of an ideal DNA G4 without loops or bulges to our structure (Figure 3). The X-ray structures with PDBIDs 244D (63) and 352D (57) each contain four tetramolecular G4s in their asymmetric units, providing up to 128 values for each torsion, thus giving a good estimate for the preferred range for each angle. We also considered the torsion angle values of the NMR ensembles PDBID 2JT7 (64) and PDBID 2KVY (65) with 10 models each that contributed another 320 values (Figure 3A). The presence of the bulge changes the γ torsion angle of dG8 to an unusual value of −60.6° (chain A) and −77.6° (chain B). dG6 has no unusual backbone torsion angles (Figure 3B, Supplementary Table S4). Strikingly, the ideal G4 is the almost completely devoid of any ζ angles in the range of 75°–180°. The existence of any ζ angles in this range is apparently correlated with the presence of loops. All observed backbone torsion angles of our hTR 1-20 structure are within the allowed range for nucleic acids, and there is no energetic penalty from strained backbone torsion angles in accommodating a bulge or loop into the G4 core.

In 2014, an NMR study (66) was published that established the parallel nature of the hTR 1-18 RNA G4 and the presence of a cytosine bulge. Like its DNA counterpart, the RNA G4 also formed a dimer and therefore, due to peak broadening, they could not obtain atomic coordinates by NMR approaches.

### Sedimentation velocity demonstrates the dimeric nature of hTR 1-20 DNA

**Figure 4.**
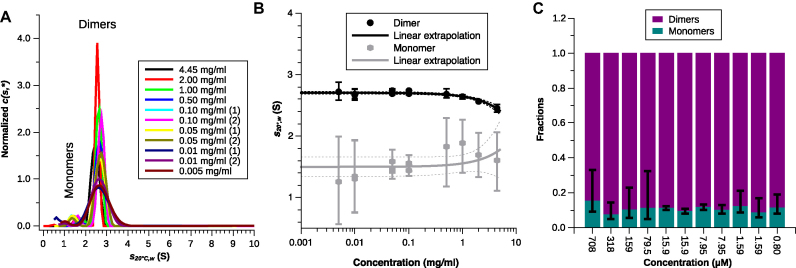
(**A**) Normalized one-dimensional *c*(s, *) distributions derived by collapsing the two-dimensional *c*(*s, f_r_*) distributions calculated from the sedimentation velocity data obtained from a wide range of concentrations (0.005–4.45 mg/ml, 0.8 μM–708 μM). Some concentrations were measured as independent duplicates. The values were corrected to standard conditions (pure water at 20°C). (**B**) Linear extrapolation to infinite dilution (solid lines) of the corrected apparent sedimentation coefficients *s_20°C,w_* obtained from direct fitting *s, M* and molecular fractions to the combined Lamm and Svedberg equations for each sample concentration. The black colour applies to the dimer and the grey colour to the monomer. *s*_20°C_*_,w_* of the dimer is decreasing with increasing concentration as would be observed for a non-interacting system; i.e. the dimer is extremely stable. To accommodate all data, we used a logarithmic scale for the abscissa. The stippled lines represent the 95% confidence intervals of the extrapolation and the error bars the 95% confidence intervals of individual data points. (**C**) Fractions of monomers (teal) and dimers (magenta) obtained from the same fits as described above. The error bars indicate the 95% confidence intervals which are determined by the noise in the absorbance data. The G4 is predominantly dimeric in the entire concentration range investigated.

We performed sedimentation velocity (SV) to study the size distributions of the hTR 1-20 DNA c2 over a wide range of concentrations (11.12–0.005 mg/ml). Due to the large absorption coefficient of DNA we could cover the entire concentration range using the absorbance optics (Figure 4A). The *c*(*s, f_r_*) distributions (Supplementary Figure S8A–M, Supplementary Table S5A-M) calculated from the sedimentation profiles suggest that we have a major and a minor peak within the sample concentration range of 4.45 to 0.005 mg/ml. The dominant peak has an *s* range of 2.45–2.73 S and a mass range 13.6–17.4 kDa, whereas the smaller peak comprises an *s* range of 1.37–1.77 S and a mass range 4.5–6.5 kDa. The *c*(*s, f_r_*) distributions of the two largest sample concentrations (11.12 and 8.90 mg/ml) contain additional features that could be due to non-ideal sedimentation imparted by the high concentration and charge of the G4s. We therefore excluded these data from further analysis. Figure 4B and C show the results of direct fitting of the apparent sedimentation constant *s*, the apparent mass *M* and the molecular fractions of the two identified species at each sample concentration to the combined Lamm and Svedberg equations. Linear extrapolation of the values to infinite dilution and conversion to standard conditions yielded an *s*^0^_20°C_*_,w_* of 2.70 ± 0.02 S and *M*^0^ of 13.5 ± 0.7 M for the larger species and an *s*^0^_20°C_*_,w_* of 1.5 ± 0.2 S and *M*^0^ of 4.7 ± 0.9 for the smaller species (Table 1). Alternatively using the peak positions of the *c*(*s, f_r_*) or *c*(*s*) distribution for the linear extrapolation yielded similar values (Table 1, Supplementary Figure S4A–E). Correlating the obtained *s*^0^_20°C_*_,w_* and *M*^0^ with the values calculated from our X-ray crystal structure in *HYDROPRO* (39–41), we could assign the major species to G4 dimers and the minor species to G4 monomers. The species analysis determined the molecular fraction of the larger species to 84.7–92.5% of the population (Figure 4C). Determining the molecular fractions by integrating the peaks of the *c*(*s, f_r_*) or *c*(*s*) distribution yielded similar values (Supplementary Figure S4G and H). These results suggest a monomer-dimer equilibrium with a large majority of dimers. There is no apparent trend of increasing monomeric fraction with decreasing sample concentration. Therefore, we could not determine the dissociation constant, but it must be lower than the lowest concentration we measured (800 nM).

### Molecular mass determination by SEC-MALS

We also measured the molecular mass of the hTR 1-20 DNA c2 by SEC-MALS using a 24 ml Superose 12 10/300 GL SEC column, see Materials and Methods. We obtained a mass of 14 ± 1 kDa at the center of the elution peak (Table 1), corroborating the value from SV and the predominant dimeric state of the G4. The resolution of the column was not enough to visually separate monomers from dimers (Supplementary Figure S5A). The molecular mass distribution shows a reduction of the mass across the elution peak indicating an increasing amount of monomer with continuing elution (Supplementary Figure S5B).

### Low resolution shape determination by small-angle X-ray scattering

**Figure 5.**
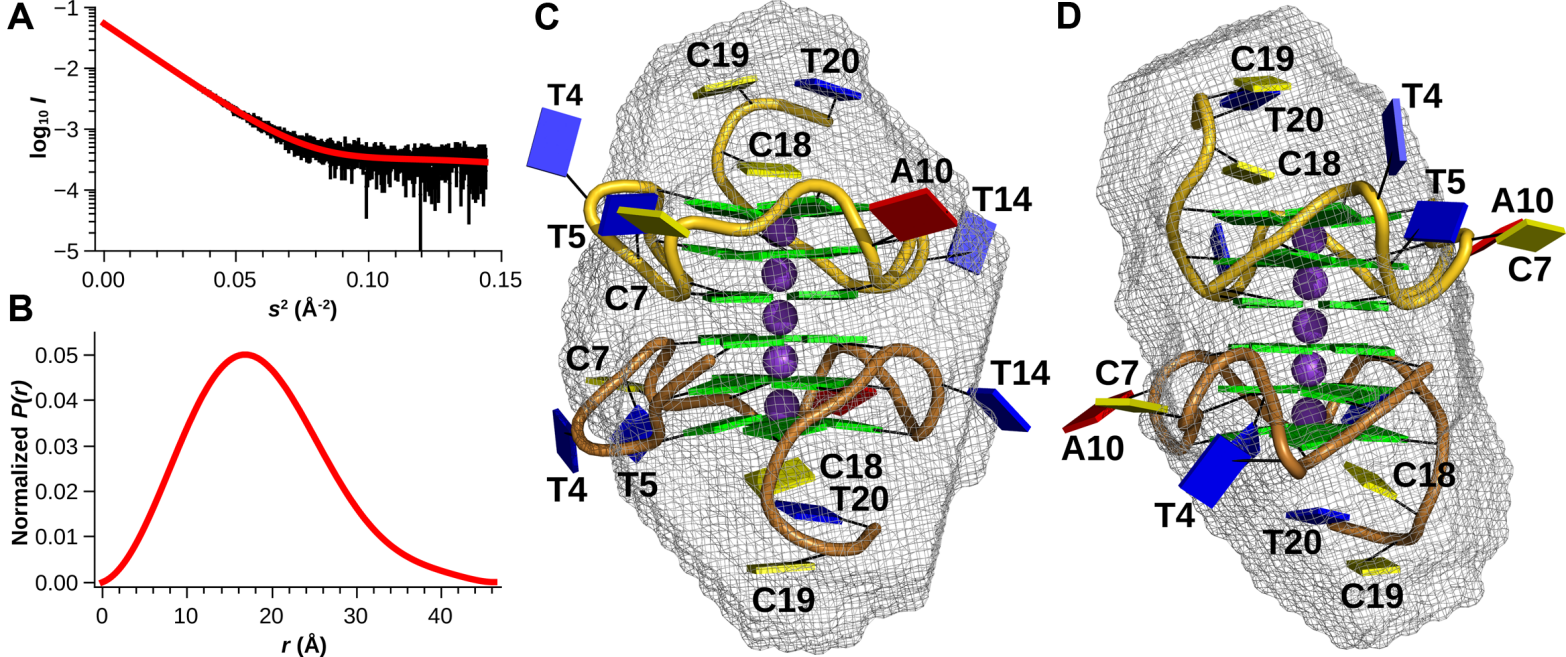
Representation of the synchrotron SEC-SAXS data. (**A**) Guinier plot of the merged scattering intensities (vertical lines represent the standard uncertainties) and the regularized curve (red line). **(B)** The pair-distance distribution *P(r)* determined from the scattering data suggest an ellipsoidal shape of the scattering hTR 1-20 DNA c2 particles with a maximal diameter of 4.6 nm. **(C and D)** Superposition of a cartoon representation of the hTR 1-20 DNA c2 X-ray crystal structure with the refined SAXS envelope from *DAMMIN* that is based on a fixed core generated from 20 averaged and filtered *DAMMIF* models using the compact-hollow shape class. (**C**) Side view of the SAXS envelope. The CCT tail and the loops with thymidylates occupy the ends of the longest axis. (**D**) 90° rotated view around the central G4 axis. The cytosine bulge and the adenylate loops are situated at the ends of the shortest axis.

In 2013, we published (18) solution structures of hTR 1-20 DNA c2 obtained by Small Angle X-ray Scattering (SAXS) that were based on data collected in batch mode (capillary) by our in-house diffractometer (Supplementary Figure S7B–D, Supplementary Table S2). To get the best possible structures, we now collected synchrotron scattering data (Figure 5A) at Beamline B21 at the Diamond Light Source (Didcot, UK) using a special flow cell that was connected to an HPLC system equipped with a 4.6 ml Shodex KW402.5-4F size exclusion column (see Materials and Methods). Synchrotron radiation offers usable data to higher angles than the home source could provide (Supplementary Figure S7A), better signal to noise, higher density of measurement points and fast data acquisition, greatly decreasing the uncertainty in the data. Passing the sample through the size exclusion column guarantees equilibrium between buffer and samples, removes aggregated material and separates oligomeric states (if their hydrodynamic volumes are sufficiently distinct) which results into more reliable models. Supplementary Figure S6A shows the scattering signal plot of the sample eluting from the SEC column. Two peaks of which the first occupies 91% and the second 9% of the total area are visible. This is consistent with the ratio of dimers to monomers that we observed by SV. However, it is improbable that the Shodex column provides enough resolution power to separate monomers and dimers. The small peak did not provide enough signal to allow further analysis, but it is most likely a baseline fluctuation.

An advantage of the synchrotron data is that the unmeasurable scattering intensity at 0° angle can be reliably determined by extrapolation and from this the molecular mass. Using the algorithm implemented in the program SAXSMOW2 (38) we obtained a molecular mass of 14 kDa (Table 1), consistent with the values of SV and in-line SEC-MALS, which independently confirms the predominantly dimeric state of our G4 in solution.

Comparing the pair-distance distribution *P*(*r*), a histogram of the inter-electron distances in the sample, of the synchrotron data to the in-house data shows the presence of an extended tail in the former that suggest that the shape should be more ellipsoidal (Figure 5B, Supplementary Figure S6B) than our previously determined models. As set out in Materials and Methods, we generated each final *DAMMIN* model based an individual fixed core calculated from a set of 20 *DAMMIF* models that only differed in the random seed. The individual cores differed by *D_max_* and the selected shape class in *DAMMIF*. For the SEC-SAXS data, we generated a total of 9 final *DAMMIN* models all of which were very symmetrical and nearly identical in shape and volume with normalized spatial discrepancies (NSDs) (34) ranging just from 0.434 to 0.504 which provides high confidence in these models. Their physical and hydrodynamic parameters can be found in Table 1. A model with a fixed core based on shape class *compact-hollow* is presented in Figure 5C and D. The SEC-SAXS structures have a similar volume as the in-house SAXS ones, but are more extended and ellipsoidal, consistent with their *P*(*r*) distribution (Supplementary Table S2). In the past few years we have accumulated three in-house datasets consisting of several concentrations each. From these, we obtain consistently bun-shaped structures with volumes ranging from 15.4–23.9 nm^3^ (Supplementary Figure S7B–D). The volume of the X-ray crystal structure is 17.2 nm^3^ according to *HYDROPRO* (Table 1). All in-house and synchrotron structures have plausible hydrodynamic properties (Supplementary Table S2) and dimensions.

The best-fit superposition of the X-ray crystal structure with the SEC-SAXS envelope oriented the central axis of the guanine tetrads ∼20° inclined from the long axis of the ellipsoid. The tail of three nucleotides at the 3′-ends of each G4 monomer are located at opposite ends of the long axis. The two loops containing thymidylates are located at opposite ends of the medium axis of the ellipsoids, which nearly coincides with the 2-fold symmetry axis of the high-resolution structure. The cytosine bulge and adjacent the adenylate loop occupy one end of the short axis whilst the other end is occupied by G-tract 4 (Figure 5 and Supplementary Figure S7A).

### Comprehensive comparison of G-quadruplexes with bulges

**Figure 6.**
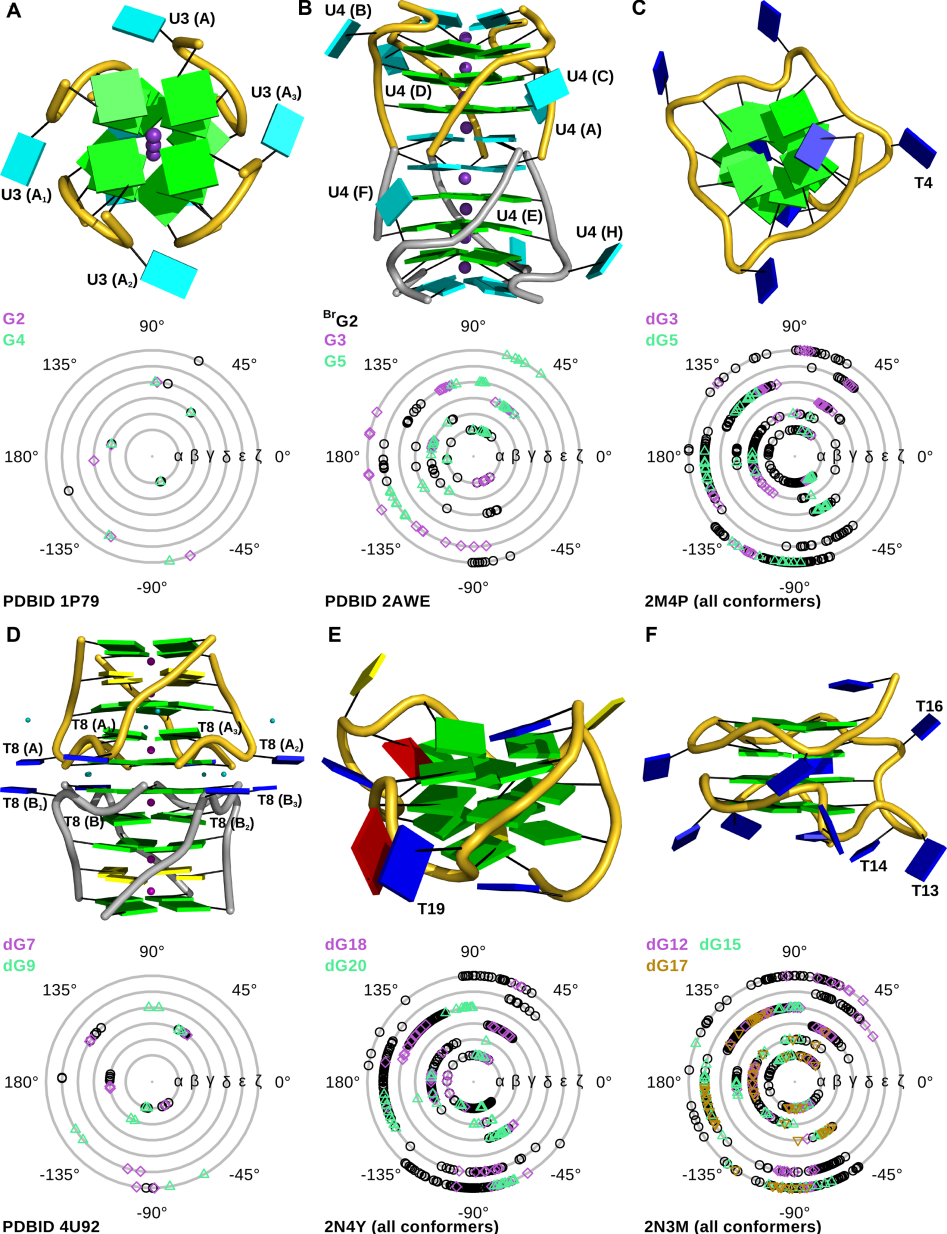
A depiction of other G4 structures in the protein data bank containing bulges rendered as cartoon (top panel) with the bases represented as blocks with following colour code: cytosines in yellow, guanines in green, thymines in blue, uracil in cyan and adenine in red. The bulges are labelled. Monomeric G4s are oriented such that the 3′-tetrad points upwards. The bottom panel shows the backbone torsion angle plot of the G4 core nucleotides. Guanylates preceding or following a bulge are marked in colour in the plot. (**A**) PDBID 1P79 (67) containing a uracil bulge (U3) in each strand. The X-ray structure is sitting on a crystallographic four-fold axis which makes each strand an exact copy (denoted A_1-3_) of strand A in the asymmetric unit and provides only 1 value for each torsion angle and nucleotide. (**B**) PDBID 2AWE (68) with one uracil bulge (U4) in each chain. This X-ray structure provides 8 values for each torsion angle and guanylate. (**C**) NMR ensemble 2M4P (2) with a single thymine bulge. (**D**) PDBID 4U92 (61) containing barium (purple) and magnesium ions (teal). This X-ray crystal structure contains two independent chains (A and B) in the asymmetric units, the other (A_1-3_, B_1-3_) are symmetric copies. This provides 2 values per torsion angle and nucleotide. (**E**) The NMR ensemble 2N4Y (69) with 10 models has one thymine bulge (T16). (**F**) NMR ensemble 2N3M (70) containing 10 models has a single thymine bulge dT16 and a double thymine bulge dT13+dT14. dG15 flanks both bulges.

In this text, we will refer to a single G4 entity in a crystal or in solution as *monomer*, regardless if it is uni-, bi- or tetramolecular, and to a stacked double G4 as *dimer*. Several parallel G4s structures with bulges have been reported so far, either with thymines (DNA) or uracils (RNA). Our high-resolution structure is unique by being the first one with a cytosine bulge. An overview of the structures together with a plot of the backbone angles of the G4 core is shown in Figure 6.

#### RNA G4 structures with uracil bulges

PDBID 1P79 (67) is a tetramolecular RNA G4 with the sequence [r(U)(d(^Br^G)r(**U**GGU)]_4_ that was solved by X-ray crystallography. U3 of all chains forms a looped-out bulge of type I. The guanylates flanking the bulge do not have any unusual backbone torsion angles (Figure 6A). As a tetramolecular G4 it has no loops and there are no ζ torsion angles in the range of 75°–180°.

An RNA G4 that contains type I and type II bulges is PDBID 2AWE (68). Pan *et al*. attributed the presence of type II bulges to the crystal packing. This G4 with sequence [r(U ^Br^GG**U**GU)]_4_ dimerized to an octaplex by intercalating the U-tetrad at the 5′-end. G3 which precedes the bulge T4 populates ζ torsion angles in the 75°–180° range. The bromidated G2 adjacent to the intercalated U tetrads occupies the γ torsion angle range −45° to −75° (Figure 6B).

#### DNA G4 structures with thymine bulges

Presently, the DNA G4s with thymine bulges in the PDB are all unimolecular. PDBID 2M4P (2) with sequence d(TTG**T**GGTGGGTGGGTGGGT) has a single type I bulge (dT4). This is an NMR ensemble of 10 models which samples the conformational space of the backbone torsion angles exhaustively (Figure 6C). As in our hTR 1–20 DNA G4, the guanylate following the bulge adopts a γ torsion angles in the range of −45° to −75° in some conformers, values absent in an ideal G4 without loops or bulges. Some core guanylates in 2M4P not adjacent to the bulge populated this γ range as well, indicating that such values can also arise in the presence of loops.

PDBID 4U92 (61) with the sequence d(CCA ^CNV^KGCG**T**GG), where ^CNV^K represents cyanovinylcarbazole, is a dimeric 3′–3′ stacked tetramolecular DNA G4 containing one thymine bulge in each chain, located close to the stacking interface (the first four nucleotides are disordered in the crystal). The 3′–3′ stacking interface is mediated by magnesium ions coordinated to four phosphate groups. The G4 has four G-tetrads with a cytosine quartet interspersed between the first and second G-tetrad. The central channel is occupied by either barium ions or water molecules, and the torsion angle plot shows no unusual values (Figure 6D).

NMR ensemble 2N4Y (69) is a parallel G4 with a single thymine bulge (T19) found in the human immunodeficiency virus-1 genome with sequence d(CTGGGCGGGACTGGGGAG**T**GGT). The backbone torsion angle plot shows that the same regions are populated as in 2M4P. The range of −45° to −75° is populated mostly, but not exclusively, by the deoxyguanylates adjacent to the bulge (Figure 6E).

The most unusual G4 we found in the PDB data bank is 2N3M (yet unpublished (70)) with sequence d(TGGTGGTGGTTG**TT**G**T**GGTGGTGGTGGT). Instead of consecutive G-tracts, the deoxyguanylates within one tetrad are connected by deoxythymidylate bridges. It also contains two bulges: One formed by the single thymine dT16 and another formed by the nucleotide twosome dT13+dT14. In this NMR ensemble consisting of 10 conformers, the core guanylates sample the same backbone torsion angle space as the classic G4s 2M4P and 2N4Y (Figure 6F, C, E). The γ torsion angles in the range of −45° to −75° are mostly occupied by the guanylates flanking the two bulges: dG12, dG15 and dG17, again confirming that this range is important to accommodate the bulges.

To our knowledge, no G4 structure containing an adenine bulge has yet been deposited in the PDB. However, they do exist and were substantiated by NMR experiments (2).

## CONCLUSIONS

In this study, we present the first high-resolution structure of a G4 with a bulged cytosine base. The electron density map of hTR 1-20 DNA c2 revealed a dimeric G4 that is stacked via the 5′-tetrads of each monomer. Analytical ultracentrifugation confirmed that the macromolecule forms a stable dimer over a large concentration range (0.8 μM to 700 μM). Its dimeric state was further corroborated by SEC-MALS and SEC-SAXS. We compared the backbone torsion angles of the guanylates forming the core of parallel G4 with bulges from the PDB to an ‘ideal’ G4 [d(TGGGGT)]_4_ without bulges or loops. The core nucleotides of G4s with loops and bulges essentially occupy the same conformational space as those from the ‘ideal’ G4. However, the presence of the loops and bulges populates an additional range of ζ torsion angles between 75° and 180° and a new region of γ torsion angles between −45° to −75°. The new γ range is occupied preferentially (but not exclusively) by core guanylates adjacent to the bulges. These extra ranges fit well into the generally observed backbone torsion angles distributions for nucleic acids (71) and do not pose a conformational barrier.

As suggested earlier (2), the range of sequences that can form G4s is much greater than the traditional G_3+_N_L1_G_3+_N_L2_G_3+_N_L3_G_3+_ consensus sequence (where G_3_ represents a triplet of guanylates and N_L_ a sequence of arbitrary length connecting the G triplets by forming a loop). It is possible to introduce interruptions into the G-tracts with the length of one or two and probably more nucleotides. These bulges together with the loops could be functionalized to introduce chemical or fluorescence probes, artificial or modified nucleotides etc. for any conceivable application. Projecting outwards from the G4 core, bulges alter the surface of the DNA, enabling specific recognition by proteins or specific targeting by small molecule drugs.

## DATA AVAILABILITY

X-ray crystal structure coordinates and structure factors have been deposited to the Protein Data Bank (3) under PDBID 5UA3. The SEC-SAXS data and structures have been deposited in the Small Angle Scattering Biological Data Bank (SASBDB) (37) under accession number SASDCC8. Deposited data will be made publicly available upon publication. To obtain the raw data for any other experiments presented in this publication, kindly contact the authors. Our R (55) script that automatically creates backbone angle plots from the output of the *DSSR* program can be downloaded from the *3DNA* forum at http://x3dna.org.

## Supplementary Material

Supplementary Data
