# Supplementary material for: Structure and hydrodynamics of a DNA G-quadruplex with a cytosine bulge
Source: Nucleic Acids Res. 2018 May 1;46(10):5319–31. doi: 10.1093/nar/gky307 (PMC6007744; doi:10.1093/nar/gky307)
Supplement: Supplementary Data [file gky307_supp.pdf]

Structure and hydrodynamics of a DNA G-Quadruplex with a cytosine bulge

Supplementary Information

Markus Meier[1,*], Aniel Moya-Torres[1], Natalie J. Krahn[1], Matthew D. McDougall[1], George L. Orriss[1], Ewan K.S. McRae[1], Evan P. Booy[1], Kevin McEleney[1], Trushar R. Patel[3,4,5], Sean A. McKenna[1,2] & Jörg Stetefeld[1,2,*]

[1]Department of Chemistry, [2]Department of Biochemistry and Medical Genetics, University of Manitoba, Winnipeg, Manitoba, R3T 2N2, Canada.

[3]Alberta RNA Research and Training Institute, Department of Chemistry and Biochemistry, University of Lethbridge, Lethbridge, Alberta, T1K 3M4, Canada

[4]DiscoveryLab, Medical Sciences Building, University of Alberta, Edmonton, Alberta, T6G 2H7, Canada.

[5]Department of Microbiology, Immunology and Infectious Diseases, Cumming School of Medicine, University of Calgary, Calgary, T2N 1N4, Alberta, Canada

* To whom correspondence should be addressed.
Phone: +1 204 474 9731
Fax: +1 204 474 7608
Email: markus.meier@umanitoba.ca, jorg.stetefeld@umanitoba.ca

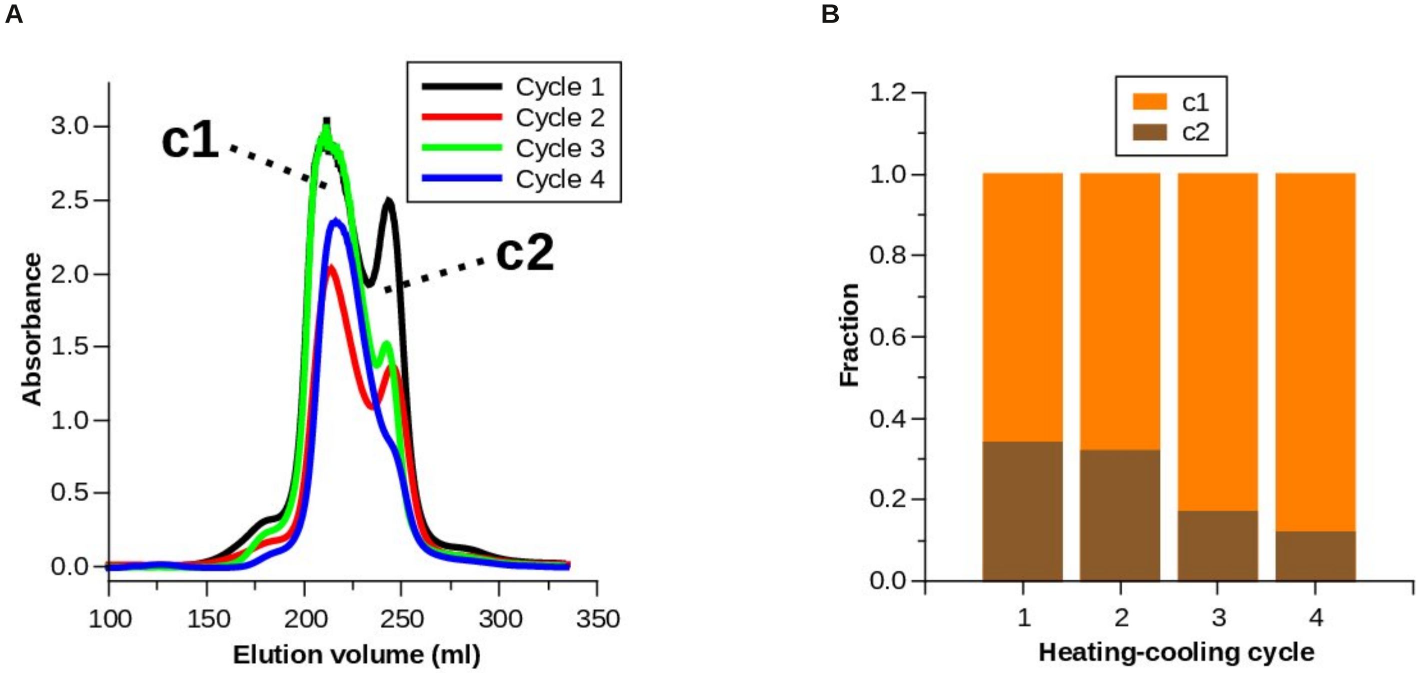

Supplementary Figure S1:
(A) Elution profiles of the hTR 1-20 DNA from the HiLoad 26/600 Superdex 75 size exclusion column. The two G4 conformations, c1 and c2, are indicated.
(B) Fractions of the G4 conformations c1 and c2 obtained in each heating and cooling cycle. The yield of conformation c2 decreased in every cycle.

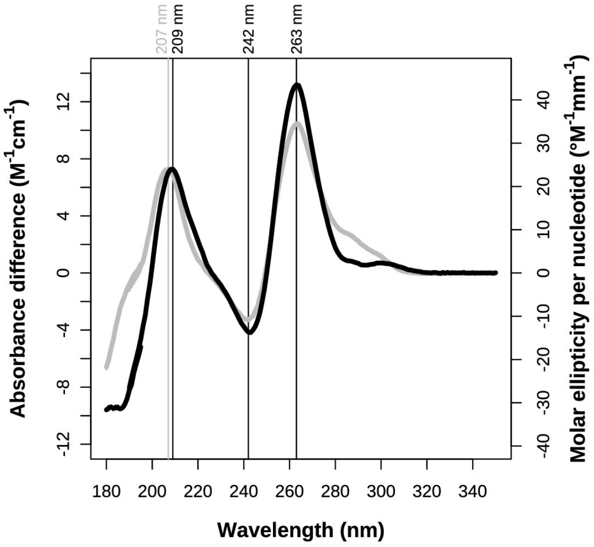

Supplementary Figure S2:
Far UV polarimetry spectra of the hTR 1-20 DNA c1 (grey) and c2 (black) recorded at 20 °C. The spectra are the average of three measurements (standard uncertainty is smaller than the line width). Maxima and minima of the spectra are indicated by vertical lines and the corresponding wavelength labels. The Figure was prepared from data published in Meier *et al.*[1] and is included here for convenience.

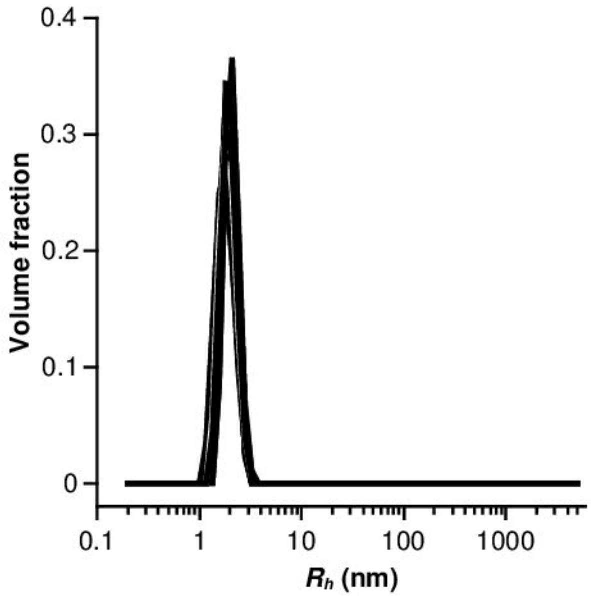

Supplementary Figure S3:
Volume weighted size distributions of the hTR 1-20 DNA c2 obtained by dynamic light scattering at 4.45 mg/ml sample concentration. Twenty measurements are shown. The sample preparation is highly pure and contains no significant amount of aggregates or higher order oligomers.

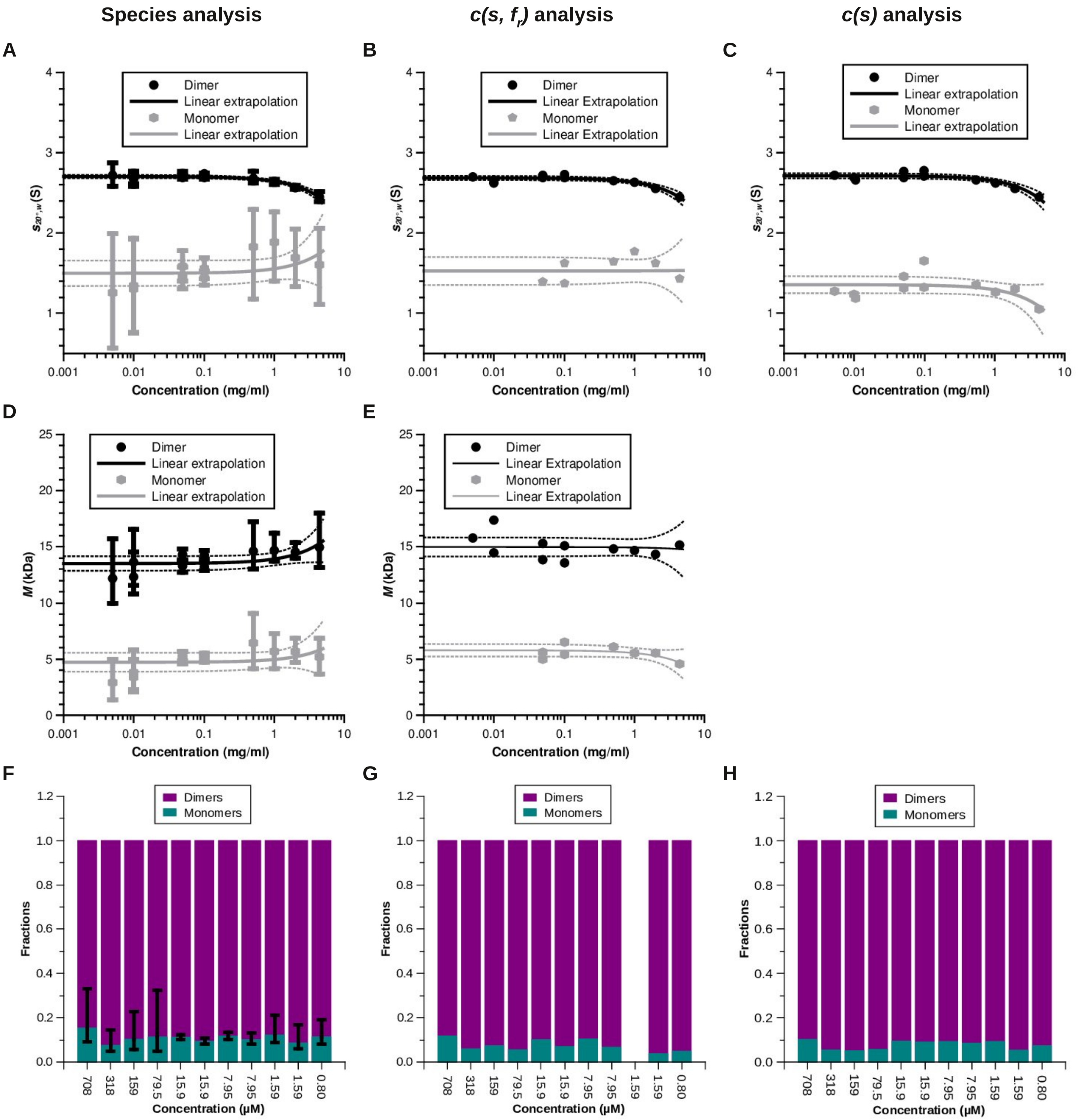

Supplementary Figure S4:

Sedimentation velocity results of the hTR 1-20 DNA c2 using the absorbance optics. **(A-C)** Linear extrapolation of sedimentation coefficient *s* of dimers (black) and monomers (grey) to infinite dilution. **(D-E)** Linear extrapolation of molar mass *M* of dimers (black) and monomers (grey) to infinite dilution. **(F-H)** Molecular fractions of dimers (magenta) and monomers (teal) in solution at different loading concentrations. The results are grouped into three columns, where the first column (A, D, F) stems from the species analysis[2], the second column (B, E, G) from the $c(s, f_r)$ analysis[3] and the last column (C, H) from the *c(s)* analysis[4] in Sedfit. Error bars indicate 95% confidence intervals of the individual data points (species analysis only). Dotted lines indicate the 95% confidence intervals of the linear extrapolation. We used a logarithmic abscissa to accommodate all concentrations in the plots. Panels (A) and (F) are identical to Fig. 4B and C in the main text and are included here for convenience.

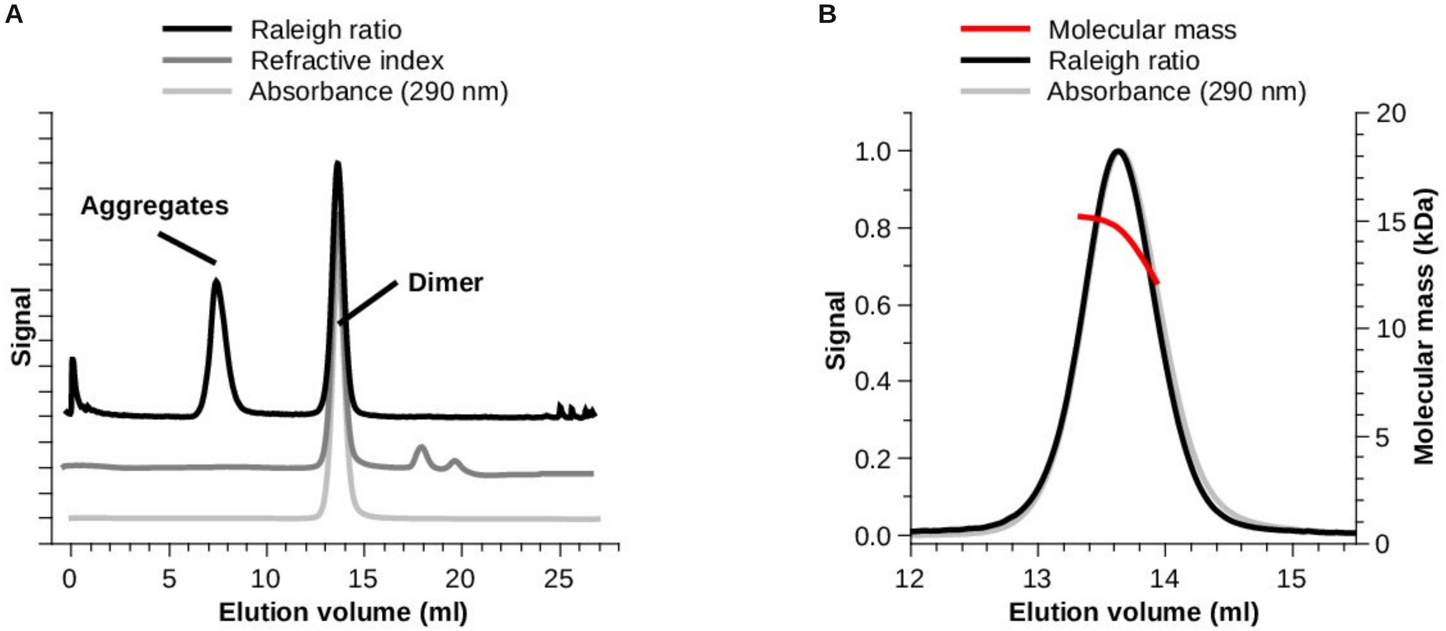

Supplementary Figure S5:
(A) SEC-MALS traces of the hTR 1-20 DNA c2 where the black line is the light scattering signal of the 90° detector, dark grey is the measured refractive index and light grey is the absorbance at 290 nm. The curves are offset for better visual separation. The 24 ml Superose 12 10/300 GL column was not able to separate monomers from dimers. A minute amount of aggregated material is present in the void volume.
(B) Determined molecular mass across the peak region (red line) based on the light scattering signals and the absorbance as concentration source.

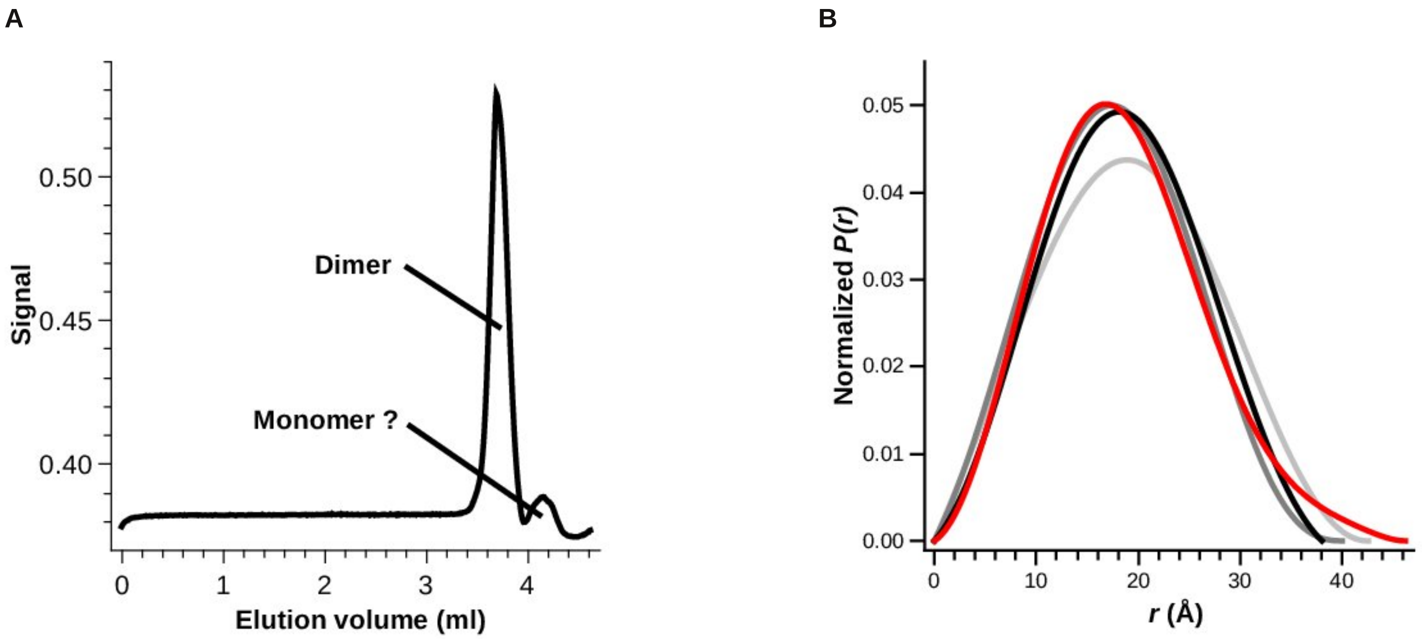

Supplementary Figure S6:
(A) Signal plot of the hTR 1-20 DNA c2 eluting from the in-line 4.6 ml Shodex KW402.5-4F size exclusion column at the B21 beamline at the Diamond Light Source. The signal is the integral of the ratio of the scattering intensity of the individual frame to the background intensity measured from the buffer. The large peak occupies 91% and the small peak 9% of the combined peak area, the same ratio that was observed for dimers and monomers by sedimentation velocity. However, the small peak is more likely baseline fluctuation, considering that the resolution power of the column is limited.
(B) Pair distance distributions obtained from independent datasets. Red: in-line SEC-SAXS collected at the Diamond Light Source; black: in-house SAXS data published in Meier *et al.*[1]; dark grey: in-house SAXS data collected in 2012; light grey: in-house SAXS data collected in 2016.

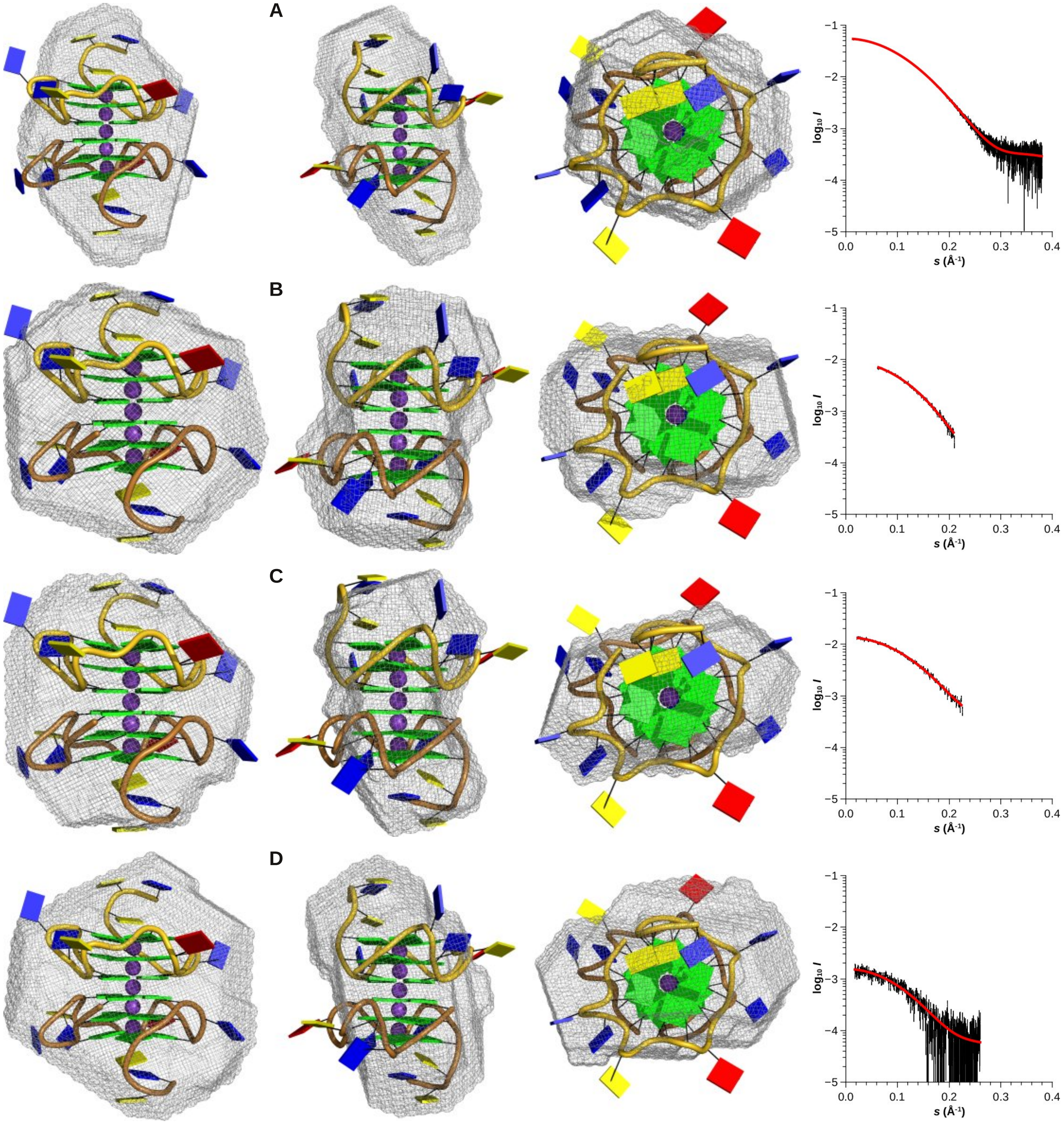

Supplementary Figure S7:
Final *DAMMIN* hTR 1-20 DNA c2 SAXS models (mesh) calculated from independent data sets viewed from front (left), side (middle) and top (right) with the high-resolution X-ray structure superimposed. The fit of each model to its data is shown at the far right graph. Each *DAMMIN* model is based on a fixed core generated from 20 averaged and filtered *DAMMIF* models.
(A) in-line SEC-SAXS data collected at the Diamond Light Source (average of 9 frames)
(B) in-house SAXS data published in Meier *et al.*[1] (average of 3 concentrations)
(C) in-house SAXS data collected in 2012 (average of 4 concentrations)
(D) in-house SAXS data collected in 2016 (average of 3 concentrations)

Supplementary Table S1: X-ray diffraction data collection and structure refinement

Parameters	Values	Details
Data reduction software	HKL2000 version 706	
Number of images	180	
Oscillation angle per image (°)	1.0	
Space group	P1	
Number of lattices	2	Stack of 2 crystals
Cell parameters (Å)	$a = 30.10 \pm 0.01$<br>$b = 33.10 \pm 0.01$<br>$c = 33.28 \pm 0.01$<br>$\alpha = 64.65 \pm 0.05$<br>$\beta = 78.64 \pm 0.05$<br>$\gamma = 81.78 \pm 0.05$	
Mosaicity range (°)	0.71 - 0.92	
Resolution (Å)	20.302 - 1.880	Cutoff: $I/\sigma_I = 2.0$
	20.302 - 1.796	Used for refinement
Number of unique measured reflections	8857	Cutoff: $I/\sigma_I = 2.0$
	9658	Used for refinement
Spherical completeness	0.960	Cutoff: $I/\sigma_I = 2.0$
	0.911	Used for refinement
Mean redundancy	3.9	Cutoff: $I/\sigma_I = 2.0$
	3.8	Used for refinement
Mean I/σ_I	9.66	Cutoff: $I/\sigma_I = 2.0$
	9.0	Used for refinement
R_{merge}	0.069	Cutoff: $I/\sigma_I = 2.0$
	0.072	Used for refinement
R_{meas}	0.081	Cutoff: $I/\sigma_I = 2.0$
	0.083	Used for refinement
R_{pim}	0.041	Cutoff: $I/\sigma_I = 2.0$
	0.042	Used for refinement
B_{Wilson} (Å ^{2})	33.5	Wilson B-factor
**Outer shell statistics for cutoff $I/\sigma_I = 2.0$**		
Spherical outer shell (Å)	1.970 - 1.880	
Number of observations	1120	unique
	4262	unmerged
Mean redundancy	3.8	

Completeness	0.927	
Mean I/σ_I	2.0	
$CC_{1/2}$	0.725	
R_{merge}	0.684	
R_{meas}	0.794	
R_{pim}	0.402	
**Outer shell statistics for processed to 1.80 Å**		
Spherical outer shell (Å)	1.910 to 1.796	
Number of observations	1173	unique
	4111	unmerged
Mean redundancy	3.5	
Completeness	0.662	
Mean I/σ_I	1.5	
$CC_{1/2}$	0.617	
R_{merge}	0.806	
R_{meas}	0.949	
R_{pim}	0.495	
**Refinement**		
R_{free}	0.247	from 603 reflections (6.8 %)
R	0.227	from 8254 reflections (93.2 %)
Estimated coordinate uncertainty (Å)	0.2	Based on *R*-value
Average *B*-factor (Å^2)	39.93	
Estimated *B*-factor uncertainty (Å^2)	9.2	Based on maximum likelihood
RMS bond lengths from ideal (Å)	0.004	
RMS bond angles from ideal (°)	1.067	
RMS chiral centre restraints (Å)	0.069	
RMS planar restraints (Å)	0.011	
Solvent content	0.644	Based on Matthews coefficient for DNA[8,9]

Supplementary Table S1: X-ray diffraction data collection and refinement statistics

Supplementary Table S2: Comparison of SAXS data and models

Dataset	Shape class	χ^2	R_g (nm)	D_{max} (nm)	V_{DAM} (nm^3)	*AER* (Å)	$R_{h\ 20°C,\ w}$ (nm)	$s_{20°C,\ w}$ (S)
in-line SEC-SAXS @ Diamond Light Source	compact-hollow	1.346	1.422	4.776	19.325	1.382	1.803	2.863
in-line SEC-SAXS @ Diamond Light Source	compact	1.346	1.422	4.880	19.259	1.382	1.802	2.864
in-line SEC-SAXS @ Diamond Light Source	unknown	1.346	1.422	4.789	19.325	1.382	1.804	2.862
Meier *et al.*[1] in-house, batch mode	unknown	0.839	1.412	4.039	19.889	1.382	1.793	2.879
2012 in-house, batch mode	unknown	1.010	1.349	3.881	15.401	1.382	1.705	3.028
2016 in-house, batch mode	unknown	1.085	1.498	4.465	23.902	1.382	1.918	2.691

Supplementary Table S2: Properties of the final *DAMMIN*[10] hTR 1-20 DNA c2 SAXS model(s) of each dataset. Each *DAMMIN* model is based on a fixed core that was generated from a set of 20 *DAMMIF*[11] models using the indicated shape class. The *DAMMIF* models in the same set were calculated with identical parameters, but different random seeds. We calculated nine different final *DAMMIN* models for the in-line synchrotron SEC-SAXS dataset by using three slightly different D_{max} values when calculating the *p(r)* distribution in *GNOM*[12] and by setting the three shape classes compact-hollow, compact and unknown in *DAMMIF*. The table shows the properties of the three SEC-SAXS models we uploaded to the SASBDB[13] under accession number SASDCC8.

R_g: Radius of gyration
D_{max}: Maximum dimension
V_{DAM}: Volume of the dummy atom model
AER: Radii of the dummy atoms (suitable for *HYDROPRO*[14-16] and visual representation)
$R_{h\ 20°C,w}$: Hydrodynamic radius in water at 20°C as calculated by *HYDROPRO*
$s_{20°C,w}$: Sedimentation coefficient in water at 20°C as calculated by *HYDROPRO*

Supplementary Table S3: Planarity of tetrads

hTR 1-20 DNA	Base pairs	Buckle (°)	Propeller Twist (°)
Tetrad 1 (A) (“planar”)	A.dG1 - A.dG6	-0.8	0.5
	A.dG1 - A.dG15	1.7	-3.3
	A.dG6 - A.dG11	1.9	-4.8
	A.dG11 - A.dG15	0.7	0.7
Tetrad 1 (B) (“planar”)	B.dG1 - B.dG6	-0.4	0.8
	B.dG1 - B.dG15	4.3	-2.5
	B.dG6 - B.dG11	2.9	-3.6
	B.dG11 - B.dG15	0.7	-0.7
Tetrad 2 (A) (“bowl”)	A.dG2 - A.dG8	3.8	9.7
	A.dG2 - A.dG16	6.7	-4.7
	A.dG8 - A.dG12	1.0	-4.7
	A.dG12 - A.dG16	3.8	4.0
Tetrad 2 (B) (“bowl”)	B.dG2 - B.dG8	5.3	8.6
	B.dG2 - B.dG16	6.8	-2.0
	B.dG8 - B.dG12	-0.1	-4.1
	B.dG12 - B.dG16	7.4	1.7
Tetrad 3 (A) (“bowl”)	A.dG3 - A.dG9	14.5	7.1
	A.dG3 - A.dG17	9.0	-0.3
	A.dG9 - A.dG13	6.9	3.4
	A.dG13 - A.dG17	10.8	5.1
Tetrad 3 (B) (“bowl”)	B.dG3 - B.dG9	17.0	5.1
	B.dG3 - B.dG17	9.6	1.6
	B.dG9 - B.dG13	11.2	4.2
	B.dG13 - B.dG17	14.2	6.7

Supplementary Table S3A: hTR 1-20 DNA c2 is an unimolecular G4 pair with 5’-5’ stacking and nucleotide sequence d(GGG TT GCGG A GGG T GGG CCT). The “buckle” and “propeller twist” base pair parameters are indicated for each tetrad.

PDBID 352D	Base pairs	Buckle (°)	Propeller Twist (°)
Tetrad 1 (ABCD) ("bowl")	A.dG2 - B.dG12	11.1	1.6
	A.dG2 - D.dG32	10.7	6.1
	B.dG12 - C.dG22	10.3	2.1
	C.dG22 - D.dG32	7.1	2.0
Tetrad 1 (EFGH) ("planar")	E.dG42 - F.dG52	-1.1	-2.0
	E.dG42 - H.dG72	-1.1	-0.9
	F.dG52 - G.dG62	-0.4	0.0
	G.dG62 - H.dG72	-0.2	-2.7
Tetrad 2 (ABCD) ("bowl")	A.dG3 - B.dG13	10.0	2.4
	A.dG3 - D.dG33	13.3	3.7
	B.dG13 - C.dG23	11.0	2.3
	C.dG23 - D.dG33	8.6	4.8
Tetrad 2 (EFGH) ("bowl")	E.dG43 - F.dG53	12.1	1.5
	E.dG43 - H.dG73	11.8	4.1
	F.dG53 - G.dG63	10.7	2.3
	G.dG63 - H.dG73	10.3	2.5
Tetrad 3 (ABCD) ("bowl")	A.dG4 - B.dG14	10.7	9.1
	A.dG4 - D.dG34	8.8	0.9
	B.dG14 - C.dG24	15.6	1.9
	C.dG24 - D.dG34	11.8	2.1
Tetrad 3 (EFGH) ("bowl")	E.dG44 - F.dG54	13.3	2.7
	E.dG44 - H.dG74	11.8	4.2
	F.dG54 - G.dG64	10.1	4.2
	G.dG64 - H.dG74	13.3	4.2
Tetrad 4 (ABCD) ("bowl")	A.dG5 - B.dG15	16.9	14.3
	A.dG5 - D.dG35	3.3	2.3
	B.dG15 - C.dG25	17.4	1.8
	C.dG25 - D.dG35	16.5	0.1
Tetrad 4 (EFGH) ("bowl")	E.dG45 - F.dG55	17.4	0.0
	E.dG45 - H.dG75	15.4	5.3
	F.dG55 - G.dG65	6.8	0.6
	G.dG65 - H.dG75	12.8	8.5

Supplementary Table S3B: PDBID 352D[17] has nucleotide sequence $[d(TGGGGT)]_4$ and is a 5'-5'-stacked tetramolecular G4 pair. From the two pairs in the asymmetric unit, we only show the base pair parameters for the ABCD/EFGH pair.

PDBID 3CCO	Base pairs	Buckle(°)	Propeller Twist(°)
Tetrad 1 (AC, BD) ("saddle")	A.dG1003 - A.dG1009	4.9	12.1
	A.dG1003 - C.dG1009	-0.6	-12.4
	A.dG1009 - C.dG1003	-0.6	-12.4
	C.dG1003 - C.dG1009	4.9	12.1
Tetrad 2 (AC, BD) ("saddle")	A.dG1004 - A.dG1010	8.2	2.8
	A.dG1004 - C.dG1010	-8.1	-5.9
	A.dG1010 - C.dG1004	-8.1	-5.9
	C.dG1004 - C.dG1010	8.2	2.8
Tetrad 3 (AC, BD) ("bowl")	A.dG1005 - A.dG1011	22.8	3.6
	A.dG1005 - C.dG1011	10.3	4.8
	A.dG1011 - C.dG1005	10.3	4.8
	C.dG1005 - C.dG1011	22.7	3.6

Supplementary Table S3C: PDBID 3CCO[18] is a pair of 5'-5'-stacked bimolecular G4s. They have nucleotide sequence $[d(TA\ GGG\ TTA\ GGG\ T)]_2$. Chains B, C, D are generated from chain A by the crystallographic symmetry operators, hence the two G4s are perfectly symmetric and have the same base-pair parameters.

PDBID 3QSF	Base pairs	Buckle (°)	Propeller Twist (°)
Tetrad 1 (XY)	X.dG1002 - X.dG1008	3.9	-10.9
	X.dG1002 - Y.dG1008	3.5	7.1
	X.dG1008 - Y.dG1002	3.5	7.1
	Y.dG1002 - Y.dG1008	3.9	-10.9
Tetrad 2 (XY) (“saddle”)	X.dG1003 - X.dG1009	-8.9	-11.7
	X.dG1003 - Y.dG1009	5.5	6.1
	X.dG1009 - Y.dG1003	5.5	6.1
	Y.dG1003 - Y.dG1009	-8.9	-11.7
Tetrad 3 (XY) (“bowl”)	X.dG1004 - X.dG1010	4.3	1.2
	X.dG1004 - Y.dG1010	16.9	3.3
	X.dG1010 - Y.dG1004	16.9	3.3
	Y.dG1004 - Y.dG1010	4.3	1.2

Supplementary Table S3D: PDBID 3QSF[19] with nucleotide sequence [d(A GGG TTA GGG TT)]$_2$ is a 5’-5’-stacked bimolecular G4 pair. Chain Y as well as the stacked partner are generated from chain X by the crystallographic symmetry operators and therefore we only show the base pair parameters for the G4 XY.

PDBID 2LE6 (model 1)	Base pairs	Buckle (°)	Propeller Twist (°)
Tetrad 1 (A)	A.dG1 - A.dG5	-1.5	1.2
	A.dG1 - A.dG13	-2.4	5.0
	A.dG5 - A.dG9	3.8	-0.3
	A.dG9 - A.dG13	3.8	7.4
Tetrad 1 (B)	B.dG1 - B.dG5	-4.1	-5.2
	B.dG1 - B.dG13	-4.6	10.4
	B.dG5 - B.dG9	3.8	3.0
	B.dG9 - B.dG13	3.4	3.3
Tetrad 2 (A)	A.dI2 - A.dG6	5.4	3.1
	A.dI2 - A.dG14	1.5	1.2
	A.dG6 - A.dG10	-7.1	0.3
	A.dG10 - A.dG14	6.2	-5.6
Tetrad 2 (B)	B.dI2 - B.dG6	-3.5	-0.8
	B.dI2 - B.dG14	5.6	1.0
	B.dG6 - B.dG10	-3.1	2.4
	B.dG10 - B.dG14	-5.0	-9.4
Tetrad 3 (A)	A.dG3 - A.dG7	4.2	3.9
	A.dG3 - A.dG15	3.4	7.9
	A.dG7 - A.dG11	-1.8	8.4
	A.dG11 - A.dG15	3.7	-1.4
Tetrad 3 (B) (“saddle”)	B.dG3 - B.dG7	-4.5	2.9
	B.dG3 - B.dG15	10.9	3.6
	B.dG7 - B.dG11	4.9	5.3
	B.dG11 - B.dG15	-6.4	-3.1

Supplementary Table S3E: PDBID 2LE6[20] with nucleotide sequence d(GIG T GGG T GGG T GGG T) is a unimolecular G4 pair with 5’-5’ stacking. Of the 10 NMR models, we show the base pair parameters of the representative model 1.

PDBID 2LEE (model 1)	Base pairs	Buckle (°)	Propeller Twist (°)
Tetrad 1 (A)	A.dG3 - A.dG7	16.3	16.7
	A.dG3 - A.dG15	12.3	2.8
	A.dG7 - A.dG11	-9.3	2.3
	A.dG11 - A.dG15	16.5	-4.6
Tetrad 2 (A) ("bowl")	A.dG4 - A.dG8	25.1	-0.8
	A.dG4 - A.dG16	11.1	-3.9
	A.dG8 - A.dG12	4.1	14.3
	A.dG12 - A.dG16	7.0	-7.7
Tetrad 3 (A)	A.dG5 - A.dG9	-3.0	-1.5
	A.dG5 - A.dG17	12.6	10.5
	A.dG9 - A.dG13	8.6	1.6
	A.dG13 - A.dG17	5.8	-5.5

Supplementary Table S3F: PDBID 2LEE[21] is a single unimolecular G4 with nucleotide sequence d(TA GGG C GGG A GGG A GGG AA). We show the base pair parameters of the first out of the 10 NMR models.

Supplementary Table S4: Backbone and sugar pucker geometry

hTR 1-20 DNA	Nucl.	α	β	γ	δ	ε	ζ	χ	Sugar Pucker
Tetrad 1	A.dG1	---	---	176.3	141.7	-162.5	-102.7	-131.0 (anti)	C2'-endo
	B.dG1	---	---	173.3	140.4	-164.8	-101.4	-135.2 (anti)	C2'-endo
	A.dG6	178.9	135.3	37.4	143.4	-90.1	-61.1	-128.4 (anti)	C2'-endo
	B.dG6	59.3	-139.5	73.9	148.7	-92.1	-51.1	-126.0 (anti)	C2'-endo
	A.dG11	60.3	-128.4	61.1	144.9	-164.1	-88.6	-129.3 (anti)	C2'-endo
	B.dG11	61.5	-129.7	59.5	142.6	-165.0	-93.1	-129.0 (anti)	C2'-endo
	A.dG15	67.9	177.7	55.0	142.2	-165.5	-93.4	-135.1 (anti)	C2'-endo
	B.dG15	58.5	-131.9	63.0	142.7	-170.1	-95.4	-130.7 (anti)	C2'-endo
Tetrad 2	A.dG2	-78.0	-163.6	41.5	147.5	-173.0	-121.4	-97.7 (anti)	C2'-endo
	B.dG2	-73.0	-166.2	43.3	140.5	-179.4	-106.7	-101.4 (anti)	C2'-endo
	A.dG8	62.1	174.7	**-60.6**	99.0	-152.5	-70.4	-176.9 (anti)	**C3'-endo**
	B.dG8	79.2	163.7	**-77.6**	106.1	-151.0	-71.1	-177.6 (anti)	**C3'-endo**
	A.dG12	-105.8	-176.6	68.7	118.8	175.7	-104.6	-118.4 (anti)	C2'-endo
	B.dG12	-96.0	-176.0	65.9	119.3	-179.9	-101.3	-117.4 (anti)	C2'-endo
	A.dG16	-82.9	-170.2	50.6	126.3	175.8	-99.0	-112.2 (anti)	C2'-endo
	B.dG16	-76.4	-162.1	42.9	137.4	177.6	-101.8	-104.7 (anti)	C2'-endo
Tetrad 3	A.dG3	-64.4	-177.9	45.2	141.1	-134.9	91.2	-108.6 (anti)	C2'-endo
	B.dG3	-64.7	-169.1	40.6	140.4	-143.6	89.2	-104.3 (anti)	C2'-endo
	A.dG9	-76.6	-160.4	64.2	156.9	-79.9	88.6	-112.6 (anti)	C2'-endo
	B.dG9	-75.8	-160.2	59.4	156.7	-75.6	80.4	-106.0 (anti)	C2'-endo
	A.dG13	-67.4	-169.2	54.5	147.4	-79.2	74.5	-105.6 (anti)	C2'-endo
	B.dG13	-63.2	-168.4	44.9	150.1	-79.3	86.8	-98.2 (anti)	C2'-endo
	A.dG17	-65.5	-163.5	44.0	138.1	-165.8	-87.5	-104.5 (anti)	C2'-endo
	B.dG17	-72.0	-164.5	46.2	137.4	-146.4	-101.4	-101.9 (anti)	C2'-endo

Supplementary Table S4: This table presents backbone torsion angles, glycosidic bond angles and sugar pucker conformations of the hTR 1-20 DNA c2 core nucleotides. The guanylates preceding and following the dC7 bulge are indicated by a red border. Interestingly, the presence of the bulge changed the sugar pucker to C3'-endo conformation in the following guanylate.

Determining the atomic element radius of the SAXS models

To verify our SAXS models, we calculated the hydrodynamic parameters (hydrodynamic radius R_h, sedimentation coefficient *s*) from the bead models using the program *HYDROPRO* (version 10)[14-16]. An important parameter for *HYDROPRO* is the *atomic element radius* (*AER*), *i.e.* the radius of the beads in the SAXS (primary) model. This parameter can be calculated from the "*dummy atom volume*" V_{DA} reported in the header of each PDB file produced by *DAMMIF*[11] by using the following equation:

$$AER = \sqrt[3]{\frac{3\,V_{DA}}{4\,\pi}}$$

The radius of gyration R_g , largest dimension D_{max}, excluded volume V_{DAM} and estimated molecular mass *M* can also be found in the PDB file header. In the case of *DAMMIN*[10] models, V_{DA} is reported in the PDB file header as "*average volume per atom*". Do **not** use "*DAM packing radius*" (*DAMMIN*) or "*dummy atom radius*" (*DAMMIF*) as *AER*, because they do not reproduce the correct V_{DAM}.

The calculated *AER* is also suitable for visual representation of the dummy atom model in PyMOL[22].

Below is an example of a set of *DAMMIF* models from our 2016 dataset:

All twenty bead models had a V_{DA} of 5.661 Å^3 that yielded an *AER* of 1.106 Å. Using this value, *HYDROPRO* calculated $V_{DAM,\ hydropro}$ = 22.7 ± 0.3 nm^3 , $R_{g,\ hydropro}$ = 1.497 ± 0.009 nm and $D_{max,\ hydropro}$ = 4.54 ± 0.02 nm. Compare this with the values from *DAMMIF*, where $V_{DAM,\ dammif}$ = 23.60 ± 0.04 nm^3 , $R_{g,\ dammif}$ = 1.4891 ± 0.0001 nm and $D_{max,\ dammif}$ = 4.52 ± 0.02 nm.

According to the *HYDROPRO* manual, the *AER* parameter must be chosen such as to faithfully reproduce the volume of the molecule. Adjusting the *AER* to 1.142 Å by trial and error reproduced V_{DAM} from *DAMMIF* very closely: $V_{DAM,\ hydropro}$ = 23.4 ± 0.3 nm^3 , $R_{g,\ hydropro}$ = 1.494 ± 0.005 nm and $D_{max,\ hydropro}$ = 4.54 ± 0.02 nm.

However, comparing the sedimentation coefficients $s_{20°C,w}$ (and hydrodynamic radii) obtained with both *AER* values, we get identical numbers ($s_{hydropro}$ = 2.72 ± 0.02 S with *AER* = 1.106 Å and $s_{hydropro}$ = 2.72 ± 0.01 S with *AER* = 1.142 Å) and they were within the experimental SV result of 2.70 ± 0.02 S (Table 1 in the associated publication). Simply calculating the *AER* parameter from V_{DA} reported by *DAMMIF* is thus the method of choice to determine the correct value for SAXS dummy atom models.

We used the atomic-level primary model calculation (INDMODE=1) with 8 shells, where the innermost shell contained 200-300 and the outermost shell 1800-3000 minibeads.

Appendix: Sedimentation velocity data analysis

11.12 mg/ml (1769 µM) hTR 1-20 DNA c2

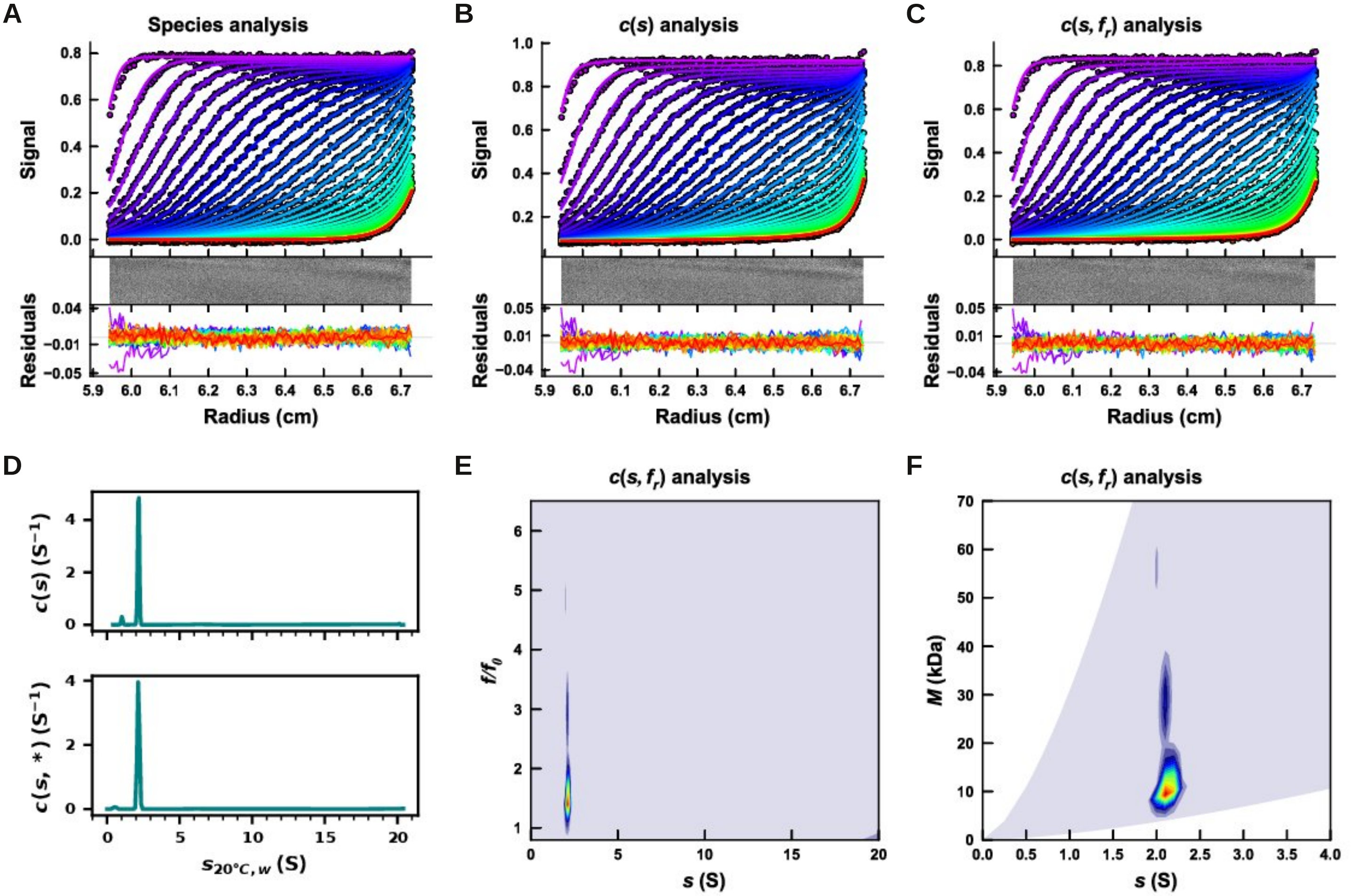

Fig. S8A: Data, fit and residuals[5] of **(A)** species analysis[2] with 2 species, **(B)** *c(s)* analysis[4] and **(C)** *c(s, f_r)* analysis[3] in Sedfit. **(D)** The top panel shows the *c(s)* distribution and the bottom panel the *c(s, *)* distribution. *s* values were corrected to standard conditions. **(E)** Plot of frictional ratio f_r versus uncorrected sedimentation coefficient *s* as obtained from *c(s, f_r)* analysis. **(F)** Plot of molecular mass *M* versus uncorrected sedimentation coefficient *s* as obtained from *c(s, f_r)* analysis.

Species	Parameter	Species analysis		*c*(*s*) analysis	*c*(*s*, f_r) analysis
	**Fit r.m.s.d.**	0.006088		0.006176	0.006018
		**Best fit value**	**95% confidence interval**		
**Species 1**	$s_{20°C,w}$ **(S)**	2.16	2.10 - 2.21	1.03	2.17
	***M* (Da)**	8150	7160 - 8980		10630
	f_r				1.51
	**Fraction**	0.661	0.526 - 0.717	0.052	n/a
**Species 2**	$s_{20°C,w}$ **(S)**	2.13	2.09 - 2.17	2.17	2.14
	***M* (Da)**	27240	20550 - 34870		28090
	f_r				2.91
	**Fraction**	0.339	0.283 - 0.474	0.948	n/a

Supplementary Table S5A: SV analysis of hTR 1-20 DNA c2 at a concentration 11.12 mg/ml (1769 µM) in 20 mM HEPES, pH 7.5, 100 mM KCl using a partial specific volume $\bar{v}$ of 0.541 cm^3/g, a solvent density ρ of 1.00450 g/cm^3 and solvent viscosity η of 0.01015 P. All sedimentation coefficients were corrected to standard conditions ($s_{20°C,w}$). Other parameters shown are the molecular mass *M*, frictional ratio f_r and the relative fractions of the two species.

Additional features beyond hTR monomers and dimers are present, most likely due to non-ideal sedimentation at this high concentration. These data were therefore omitted from the global analysis.

8.90 mg/ml (1416 µM) hTR 1-20 DNA c2

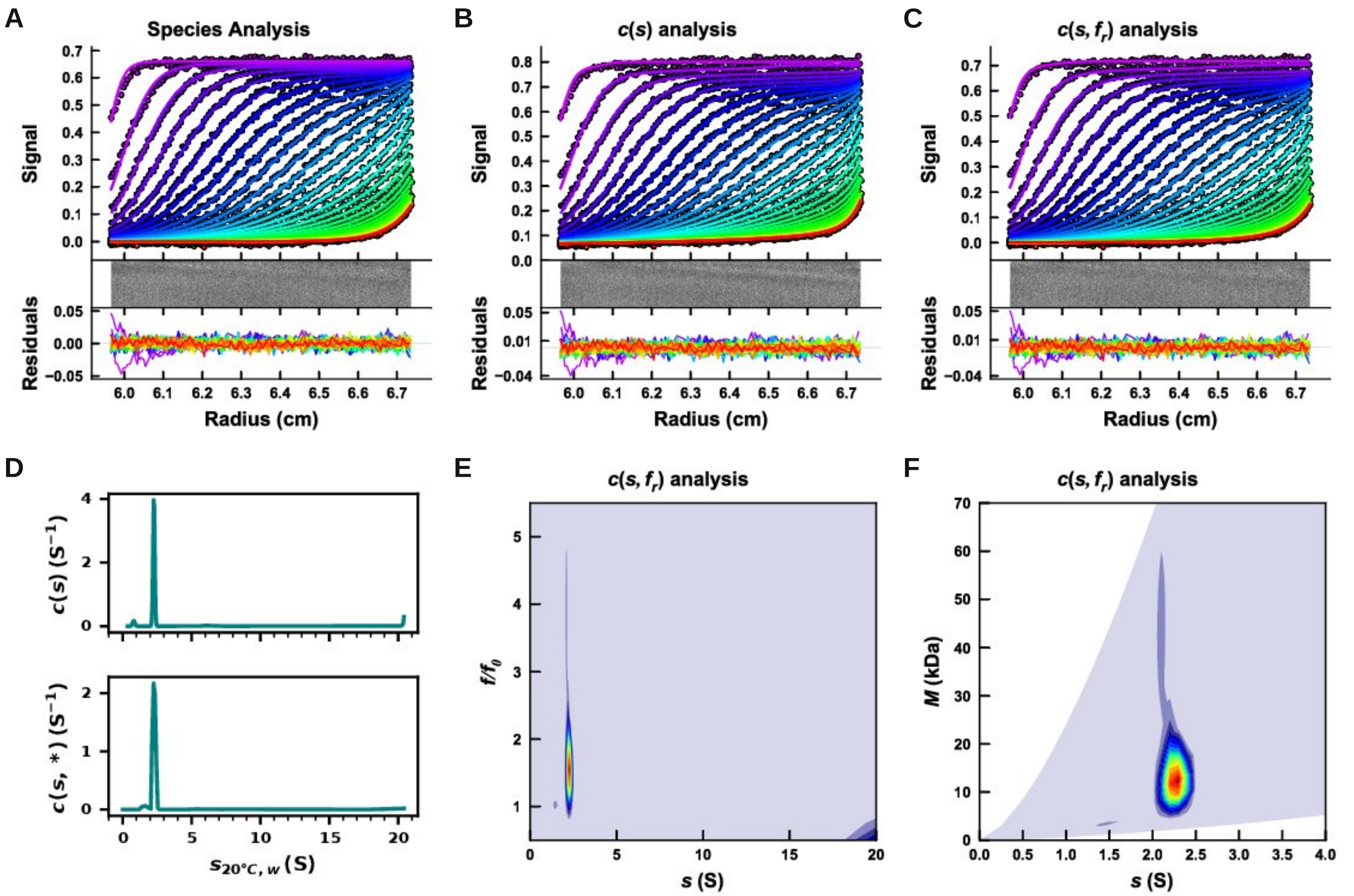

Fig. S8B: Data, fit and residuals[5] of **(A)** species analysis[2] with 2 species, **(B)** *c(s)* analysis[4] and **(C)** *c(s, f_r)* analysis[3] in Sedfit. **(D)** The top panel shows the *c(s)* distribution and the bottom panel the *c(s, *)* distribution. *s* values were corrected to standard conditions. **(E)** Plot of frictional ratio f_r versus uncorrected sedimentation coefficient *s* as obtained from *c(s, f_r)* analysis. **(F)** Plot of molecular mass *M* versus uncorrected sedimentation coefficient *s* as obtained from *c(s, f_r)* analysis.

Species	Parameter	Species analysis		*c*(*s*) analysis	*c*(*s*, f_r) analysis
	**Fit r.m.s.d.**	0.006193		0.006214	0.005998
		**Best fit value**	**95% confidence interval**		
**Species 1**	$s_{20°C,w}$ **(S)**	2.31	2.22 - 2.43	0.80	1.51
	M **(Da)**	6810	5540 - 8380		3480
	f_r				1.03
	**Fraction**	0.494	0.355 - 0.616	0.052	0.047
**Species 2**	$s_{20°C,w}$ **(S)**	2.20	2.15 - 2.24	2.27	2.30
	M **(Da)**	22100	17700 - 26690		12690
	f_r				1.60
	**Fraction**	0.506	0.384 - 0.645	0.948	0.953

Supplementary Table S5B: SV analysis of hTR 1-20 DNA c2 at a concentration 8.90 mg/ml (1416 µM) in 20 mM HEPES, pH 7.5, 100 mM KCl using a partial specific volume $\bar{v}$ of 0.541 cm^3/g, a solvent density ρ of 1.00450 g/cm^3 and solvent viscosity η of 0.01015 P. All sedimentation coefficients were corrected to standard conditions ($s_{20°C,w}$). Other parameters shown are the molecular mass *M*, frictional ratio f_r and the relative fractions of the two species.

Additional features beyond hTR monomer and dimers are present, most likely due to non-ideal sedimentation at this high concentration. These data were therefore omitted from the global analysis.

4.45 mg/ml (708 µM) hTR 1-20 DNA c2

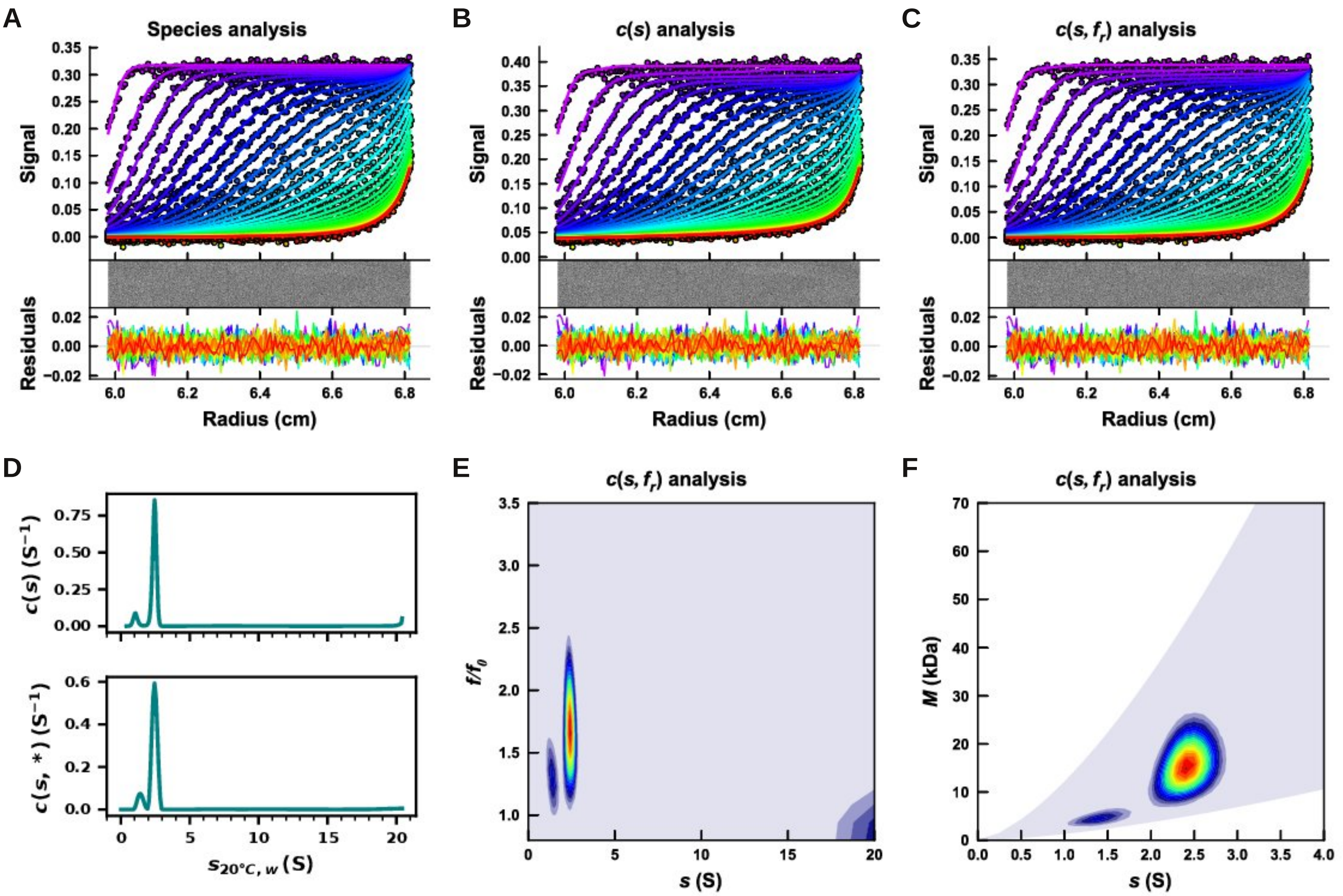

Fig. S8C: Data, fit and residuals[5] of **(A)** species analysis[2] with 2 species, **(B)** *c(s)* analysis[4] and **(C)** *c(s, f_r)* analysis[3] in Sedfit. **(D)** The top panel shows the *c(s)* distribution and the bottom panel the *c(s, *)* distribution. *s* values were corrected to standard conditions. **(E)** Plot of frictional ratio f_r versus uncorrected sedimentation coefficient *s* as obtained from *c(s, f_r)* analysis. **(F)** Plot of molecular mass *M* versus uncorrected sedimentation coefficient *s* as obtained from *c(s, f_r)* analysis.

Species	Parameter	Species analysis		*c*(*s*) analysis	*c*(*s*, f_r) analysis
	**Fit r.m.s.d.**	0.005189		0.005204	0.005171
		**Best fit value**	**95% confidence interval**		
**Monomer**	$s_{20°C,w}$ **(S)**	1.60	1.10 - 2.07	1.05	1.43
	M **(Da)**	5210	3660 - 6860		4540
	f_r				1.30
	**Fraction**	0.153	0.09 - 0.331	0.102	0.117
**Dimer**	$s_{20°C,w}$ **(S)**	2.45	2.39 - 2.53	2.45	2.45
	M **(Da)**	14920	13140 - 17980		15150
	f_r				1.69
	**Fraction**	0.847	0.669 - 0.910	0.898	0.883

Supplementary Table S5C: SV analysis of hTR 1-20 DNA c2 at a concentration 4.45 mg/ml (708 µM) in 20 mM HEPES, pH 7.5, 100 mM KCl using a partial specific volume $\bar{v}$ of 0.541 cm^3/g, a solvent density ρ of 1.00450 g/cm^3 and solvent viscosity η of 0.01015 P. All sedimentation coefficients were corrected to standard conditions ($s_{20°C,w}$). Other parameters shown are the molecular mass *M*, frictional ratio f_r and the relative fractions of the two species.

2.00 mg/ml (318 µM) hTR 1-20 DNA c2

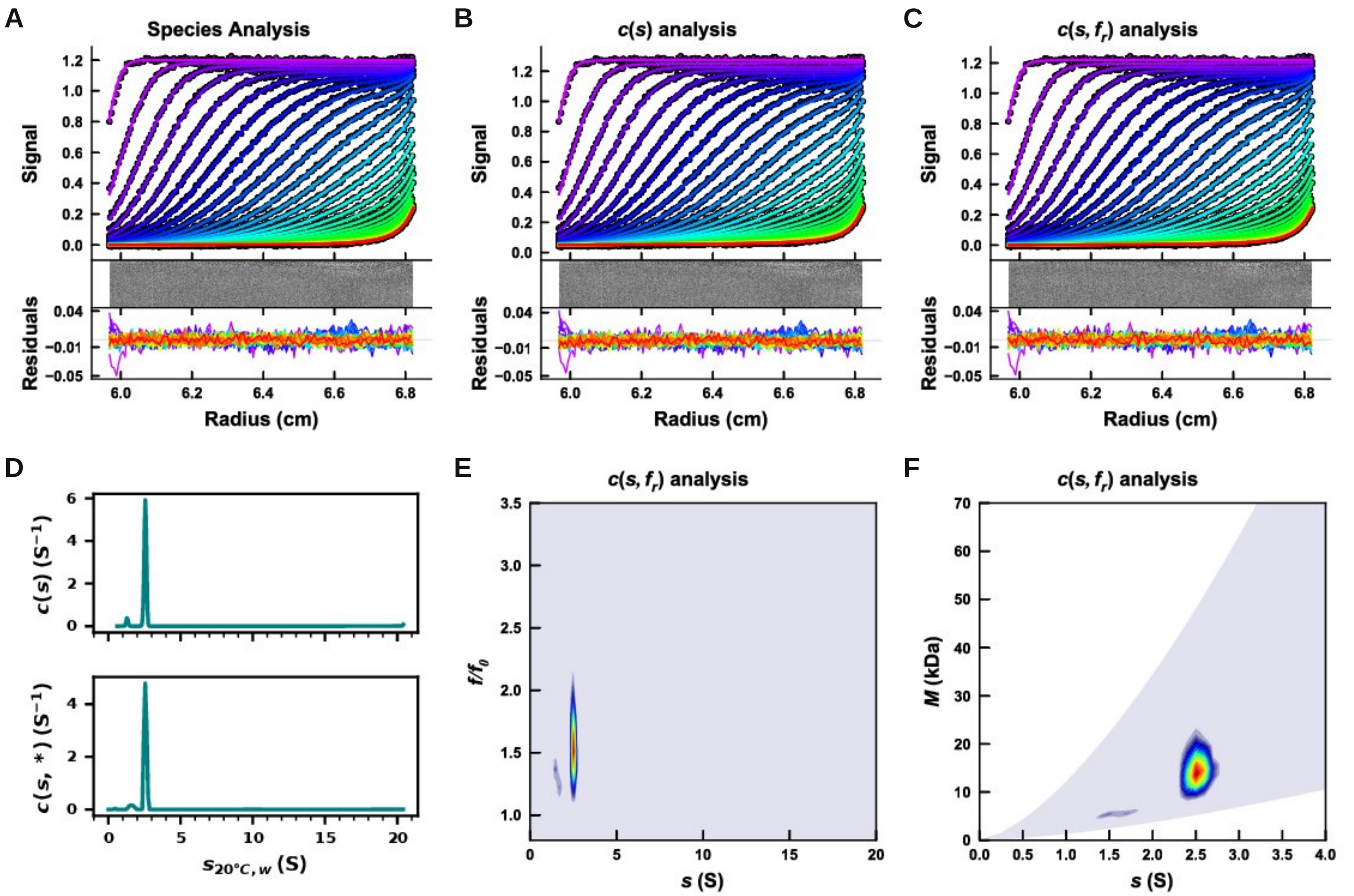

Fig. S8D: Data, fit and residuals[5] of **(A)** species analysis[2] with 2 species, **(B)** *c(s)* analysis[4] and **(C)** *c(s, f_r)* analysis[3] in Sedfit. **(D)** The top panel shows the *c(s)* distribution and the bottom panel the *c(s, *)* distribution. *s* values were corrected to standard conditions. **(E)** Plot of frictional ratio f_r versus uncorrected sedimentation coefficient *s* as obtained from *c(s, f_r)* analysis. **(F)** Plot of molecular mass *M* versus uncorrected sedimentation coefficient *s* as obtained from *c(s, f_r)* analysis.

Species	Parameter	Species analysis		*c*(*s*) analysis	*c*(*s*, f_r) analysis
	**Fit r.m.s.d.**	0.005777		0.005844	0.005806
		**Best fit value**	**95% confidence interval**		
**Monomer**	$s_{20°C,w}$ **(S)**	1.70	1.33 - 2.06	1.30	1.62
	M **(Da)**	5700	4730 - 6880		5520
	f_r				1.30
	**Fraction**	0.075	0.049 - 0.143	0.054	0.059
**Dimer**	$s_{20°C,w}$ **(S)**	2.57	2.54 - 2.60	2.56	2.56
	M **(Da)**	14550	14010 - 15370		14360
	f_r				1.56
	**Fraction**	0.925	0.857 - 0.951	0.946	0.941

Supplementary Table S5D: SV analysis of hTR 1-20 DNA c2 at a concentration 2.00 mg/ml (318 µM) in 20 mM HEPES, pH 7.5, 100 mM KCl using a partial specific volume $\bar{\nu}$ of 0.541 cm^3/g, a solvent density ρ of 1.00450 g/cm^3 and solvent viscosity η of 0.01015 P. All sedimentation coefficients were corrected to standard conditions ($s_{20°C,w}$). Other parameters shown are the molecular mass *M*, frictional ratio f_r and the relative fractions of the two species.

1.00 mg/ml (159 µM) hTR 1-20 DNA c2

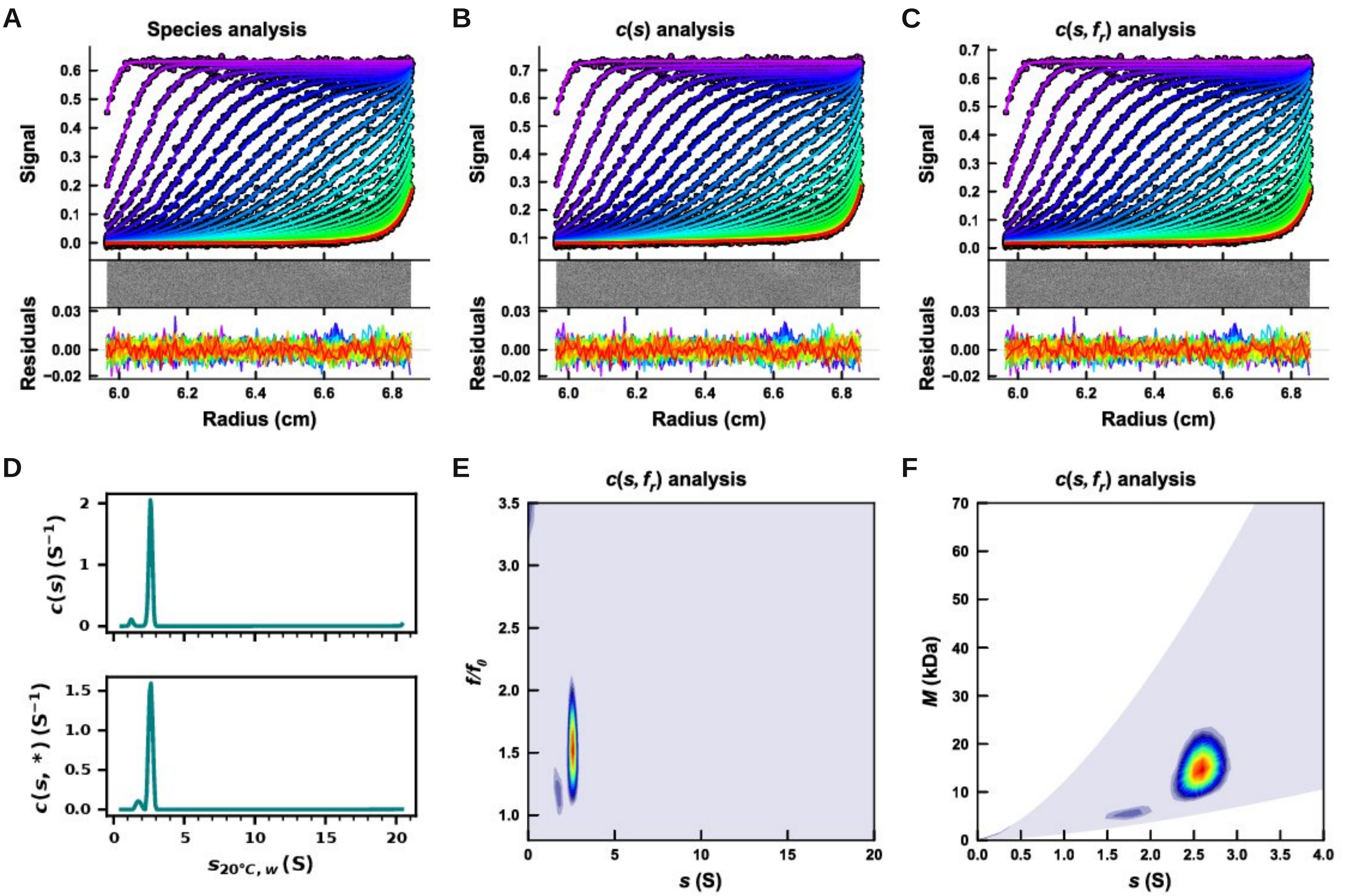

Fig. S8E: Data, fit and residuals[5] of **(A)** species analysis[2] with 2 species, **(B)** *c(s)* analysis[4] and **(C)** *c(s, f_r)* analysis[3] in Sedfit. **(D)** The top panel shows the *c(s)* distribution and the bottom panel the *c(s, *)* distribution. *s* values were corrected to standard conditions. **(E)** Plot of frictional ratio f_r versus uncorrected sedimentation coefficient *s* as obtained from *c(s, f_r)* analysis. **(F)** Plot of molecular mass *M* versus uncorrected sedimentation coefficient *s* as obtained from *c(s, f_r)* analysis.

Species	Parameter	Species analysis		*c*(*s*) analysis	*c*(*s*, f_r) analysis
	**Fit r.m.s.d.**	0.005114		0.005182	0.005101
		**Best fit value**	**95% confidence interval**		
**Monomer**	$s_{20°C,w}$ **(S)**	1.88	1.39 - 2.26	1.26	1.77
	M **(Da)**	5710	4160 - 7300		5540
	f_r				1.20
	**Fraction**	0.103	0.055 - 0.229	0.051	0.074
**Dimer**	$s_{20°C,w}$ **(S)**	2.64	2.60 - 2.68	2.62	2.63
	M **(Da)**	14680	13690 - 16200		14680
	f_r				1.54
	**Fraction**	0.897	0.771 - 0.945	0.949	0.926

Supplementary Table S5E: SV analysis of hTR 1-20 DNA c2 at a concentration 1.00 mg/ml (159 µM) in 20 mM HEPES, pH 7.5, 100 mM KCl using a partial specific volume $\bar{\nu}$ of 0.541 cm^3/g, a solvent density ρ of 1.00450 g/cm^3 and solvent viscosity η of 0.01015 P. All sedimentation coefficients were corrected to standard conditions ($s_{20°C,w}$). Other parameters shown are the molecular mass *M*, frictional ratio f_r and the relative fractions of the two species.

0.500 mg/ml (79.5 µM) hTR 1-20 DNA c2

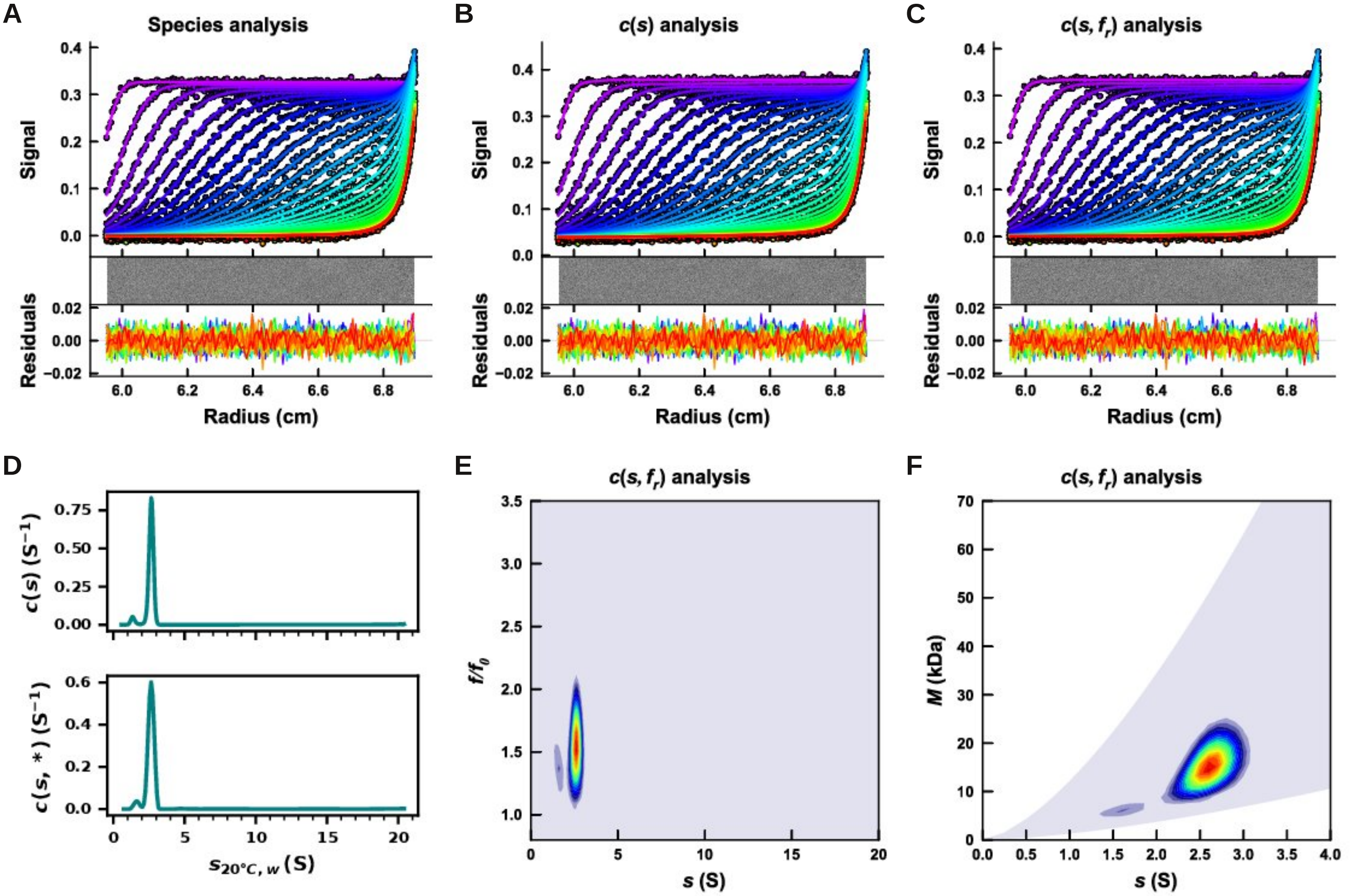

Fig. S8F: Data, fit and residuals[5] of **(A)** species analysis[2] with 2 species, **(B)** *c(s)* analysis[4] and **(C)** *c(s, f_r)* analysis[3] in Sedfit. **(D)** The top panel shows the *c(s)* distribution and the bottom panel the *c(s, *)* distribution. *s* values were corrected to standard conditions. **(E)** Plot of frictional ratio f_r versus uncorrected sedimentation coefficient *s* as obtained from *c(s, f_r)* analysis. **(F)** Plot of molecular mass *M* versus uncorrected sedimentation coefficient *s* as obtained from *c(s, f_r)* analysis.

Species	Parameter	Species analysis		*c*(*s*) analysis	*c*(*s*, f_r) analysis
	**Fit r.m.s.d.**	0.004646		0.004673	0.004657
		**Best fit value**	**95% confidence interval**		
**Monomer**	$s_{20°C,w}$ **(S)**	1.83	1.17 - 2.30	1.35	1.64
	***M* (Da)**	6470	4140 - 9050		6120
	f_r				1.37
	**Fraction**	0.113	0.050 - 0.325	0.057	0.055
**Dimer**	$s_{20°C,w}$ **(S)**	2.68	2.62 - 2.78	2.66	2.65
	***M* (Da)**	14600	13010 - 17280		14800
	f_r				1.54
	**Fraction**	0.887	0.675 - 0.950	0.943	0.945

Supplementary Table S5F: SV analysis of hTR 1-20 DNA_c2 at a concentration 0.500 mg/ml (79.5 µM) in 20 mM HEPES, pH 7.5, 100 mM KCl using a partial specific volume $\bar{\nu}$ of 0.541 cm^3/g, a solvent density ρ of 1.00450 g/cm^3 and solvent viscosity η of 0.01015 P. All sedimentation coefficients were corrected to standard conditions ($s_{20°C,w}$). Other parameters shown are the molecular mass *M*, frictional ratio f_r and the relative fractions of the two species.

Experiment #1 @ 0.100 mg/ml (15.9 µM) hTR 1-20 DNA c2

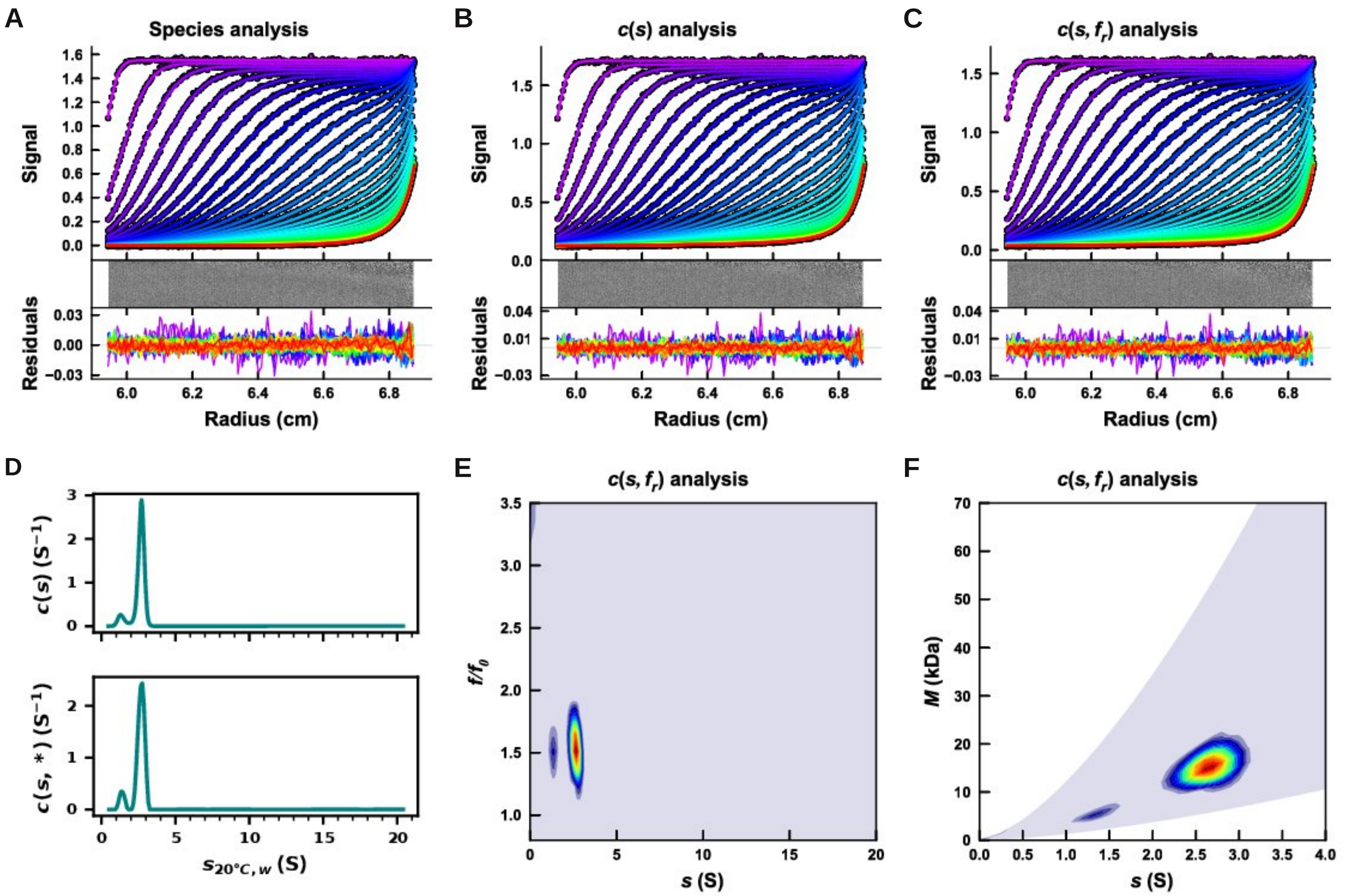

Fig. S8G: Data, fit and residuals[5] of **(A)** species analysis[2] with 2 species, **(B)** *c(s)* analysis[4] and **(C)** *c(s, f_r)* analysis[3] in Sedfit. **(D)** The top panel shows the *c(s)* distribution and the bottom panel the *c(s, *)* distribution. *s* values were corrected to standard conditions. **(E)** Plot of frictional ratio f_r versus uncorrected sedimentation coefficient *s* as obtained from *c(s, f_r)* analysis. **(F)** Plot of molecular mass *M* versus uncorrected sedimentation coefficient *s* as obtained from *c(s, f_r)* analysis.

Species	Parameter	Species analysis		*c*(*s*) analysis	*c*(*s*, f_r) analysis
	**Fit r.m.s.d.**	0.005304		0.005194	0.005284
		**Best fit value**	**95% confidence interval**		
**Monomer**	$s_{20°C,w}$ **(S)**	1.44	1.35 - 1.54	1.32	1.37
	M **(Da)**	5240	4960 - 5550		5420
	f_r				1.52
	**Fraction**	0.112	0.102 - 0.124	0.094	0.101
**Dimer**	$s_{20°C,w}$ **(S)**	2.69	2.67 - 2.71	2.71	2.69
	M **(Da)**	14280	13930 - 14690		15110
	f_r				1.54
	**Fraction**	0.888	0.876 - 0.898	0.906	0.899

Supplementary Table S5G: SV analysis of hTR 1-20 DNA_c2 at a concentration 0.100 mg/ml (15.9 µM) in 20 mM HEPES, pH 7.5, 100 mM KCl using a partial specific volume $\bar{\nu}$ of 0.541 cm^3/g, a solvent density ρ of 1.00450 g/cm^3 and solvent viscosity η of 0.01015 P. All sedimentation coefficients were corrected to standard conditions ($s_{20°C,w}$). Other parameters shown are the molecular mass *M*, frictional ratio f_r and the relative fractions of the two species.

Experiment # 2 @ 0.100 mg/ml (15.9 µM) hTR 1-20 DNA c2

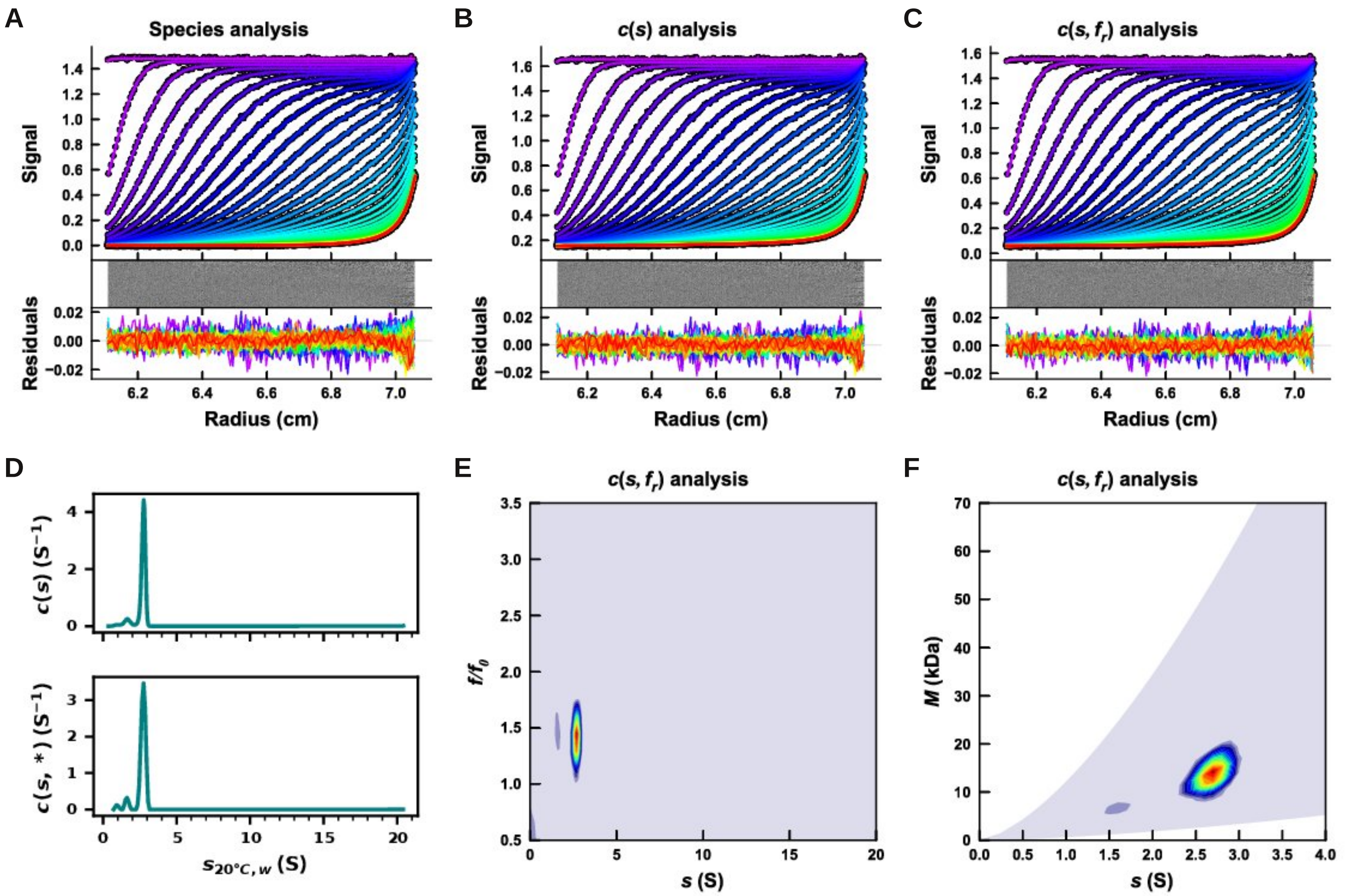

Fig. S8H: Data, fit and residuals[5] of **(A)** species analysis[2] with 2 species, **(B)** *c(s)* analysis[4] and **(C)** *c(s, f_r)* analysis[3] in Sedfit. **(D)** The top panel shows the *c(s)* distribution and the bottom panel the *c(s, *)* distribution. *s* values were corrected to standard conditions. **(E)** Plot of frictional ratio f_r versus uncorrected sedimentation coefficient *s* as obtained from *c(s, f_r)* analysis. **(F)** Plot of molecular mass *M* versus uncorrected sedimentation coefficient *s* as obtained from *c(s, f_r)* analysis.

Species	Parameter	Species analysis		*c*(*s*) analysis	*c*(*s*, f_r) analysis
	Fit r.m.s.d.	0.004722		0.004607	0.004683
		Best fit value	95% confidence interval		
Monomer	$s_{20°C,w}$ (S)	1.56	1.43 - 1.69	1.65	1.62
	M (Da)	5050	4700 - 5430		6520
	f_r				1.45
	Fraction	0.094	0.081 - 0.111	0.090	0.070
Dimer	$s_{20°C,w}$ (S)	2.74	2.72 - 2.76	2.78	2.73
	M (Da)	13240	12910 - 13630		13570
	f_r				1.41
	Fraction	0.906	0.889 - 0.919	0.910	0.930

Supplementary Table S5H: SV analysis of hTR 1-20 DNA_c2 at a concentration 0.100 mg/ml (15.9 µM) in 20 mM HEPES, pH 7.5, 100 mM KCl using a partial specific volume $\bar{\nu}$ of 0.541 cm^3/g, a solvent density ρ of 1.00450 g/cm^3 and solvent viscosity η of 0.01015 P. All sedimentation coefficients were corrected to standard conditions ($s_{20°C,w}$). Other parameters shown are the molecular mass *M*, frictional ratio f_r and the relative fractions of the two species.

Experiment # 1 @ 0.050 mg/ml (8.0 µM) hTR 1-20 DNA c2

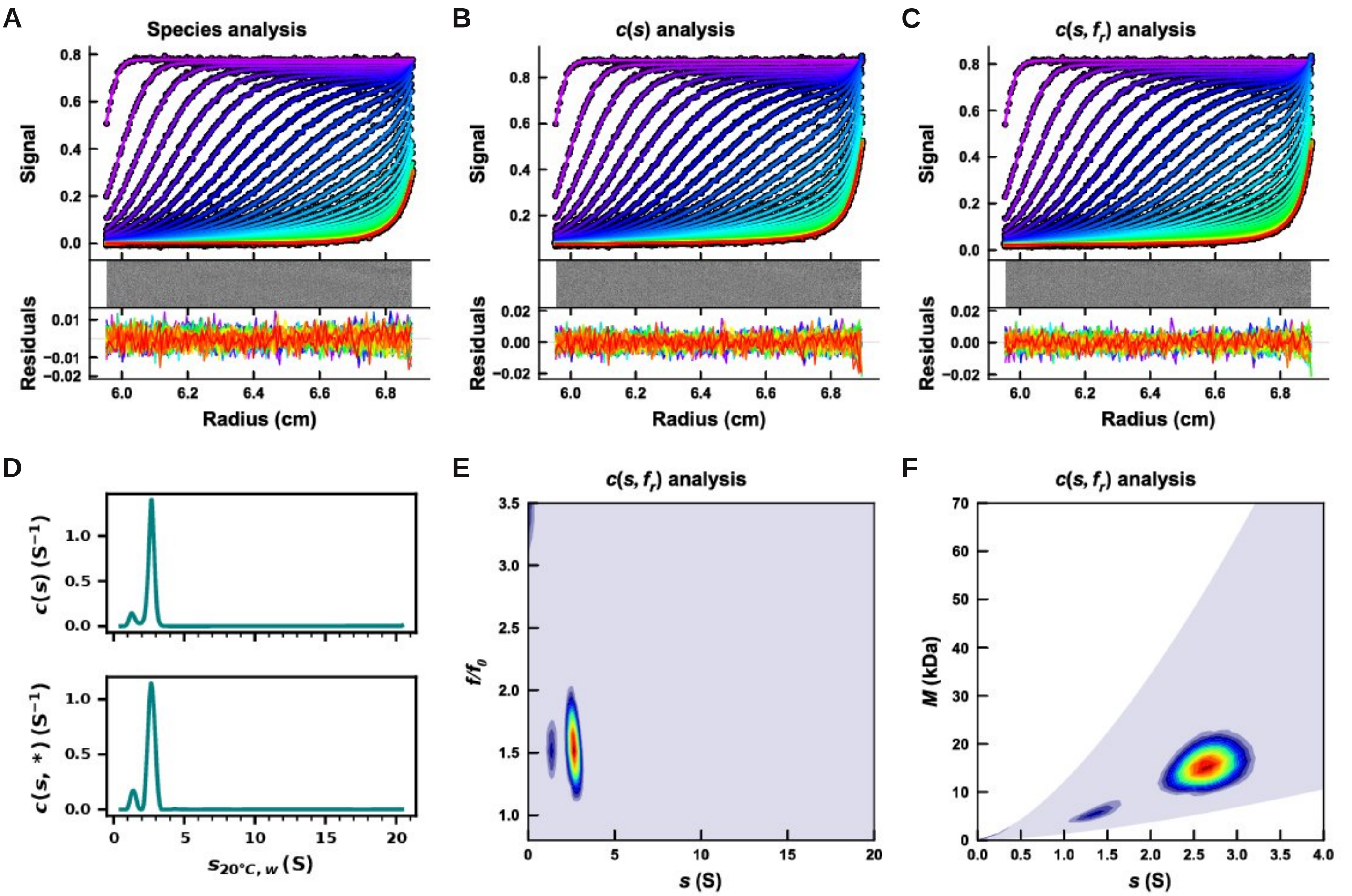

Fig. S8I: Data, fit and residuals[5] of **(A)** species analysis[2] with 2 species, **(B)** *c(s)* analysis[4] and **(C)** *c(s, f_r)* analysis[3] in Sedfit. **(D)** The top panel shows the *c(s)* distribution and the bottom panel the *c(s, *)* distribution. *s* values were corrected to standard conditions. **(E)** Plot of frictional ratio f_r versus uncorrected sedimentation coefficient *s* as obtained from *c(s, f_r)* analysis. **(F)** Plot of molecular mass *M* versus uncorrected sedimentation coefficient *s* as obtained from *c(s, f_r)* analysis.

Species	Parameter	Species analysis		*c*(*s*) analysis	*c*(*s*, f_r) analysis
	**Fit r.m.s.d.**	0.003899		0.003944	0.003931
		**Best fit value**	**95% confidence interval**		
**Monomer**	$s_{20°C,w}$ **(S)**	1.45	1.30 - 1.58	1.31	1.39
	M **(Da)**	5310	4850 - 5690		5630
	f_r				1.54
	**Fraction**	0.117	0.100 - 0.135	0.092	0.104
**Dimer**	$s_{20°C,w}$ **(S)**	2.69	2.66 - 2.71	2.69	2.69
	M **(Da)**	14310	13730 - 14850		15290
	f_r				1.55
	**Fraction**	0.883	0.865 - 0.900	0.908	0.896

Supplementary Table S5I: SV analysis of hTR 1-20 DNA c2 at a concentration 0.050 mg/ml (8.0 µM) in 20 mM HEPES, pH 7.5, 100 mM KCl using a partial specific volume $\bar{\nu}$ of 0.541 cm^3/g, a solvent density ρ of 1.00450 g/cm^3 and solvent viscosity η of 0.01015 P. All sedimentation coefficients were corrected to standard conditions ($s_{20°C,w}$). Other parameters shown are the molecular mass *M*, frictional ratio f_r and the relative fractions of the two species.

Experiment # 2 @ 0.050 mg/ml (8.0 µM) hTR 1-20 DNA c2

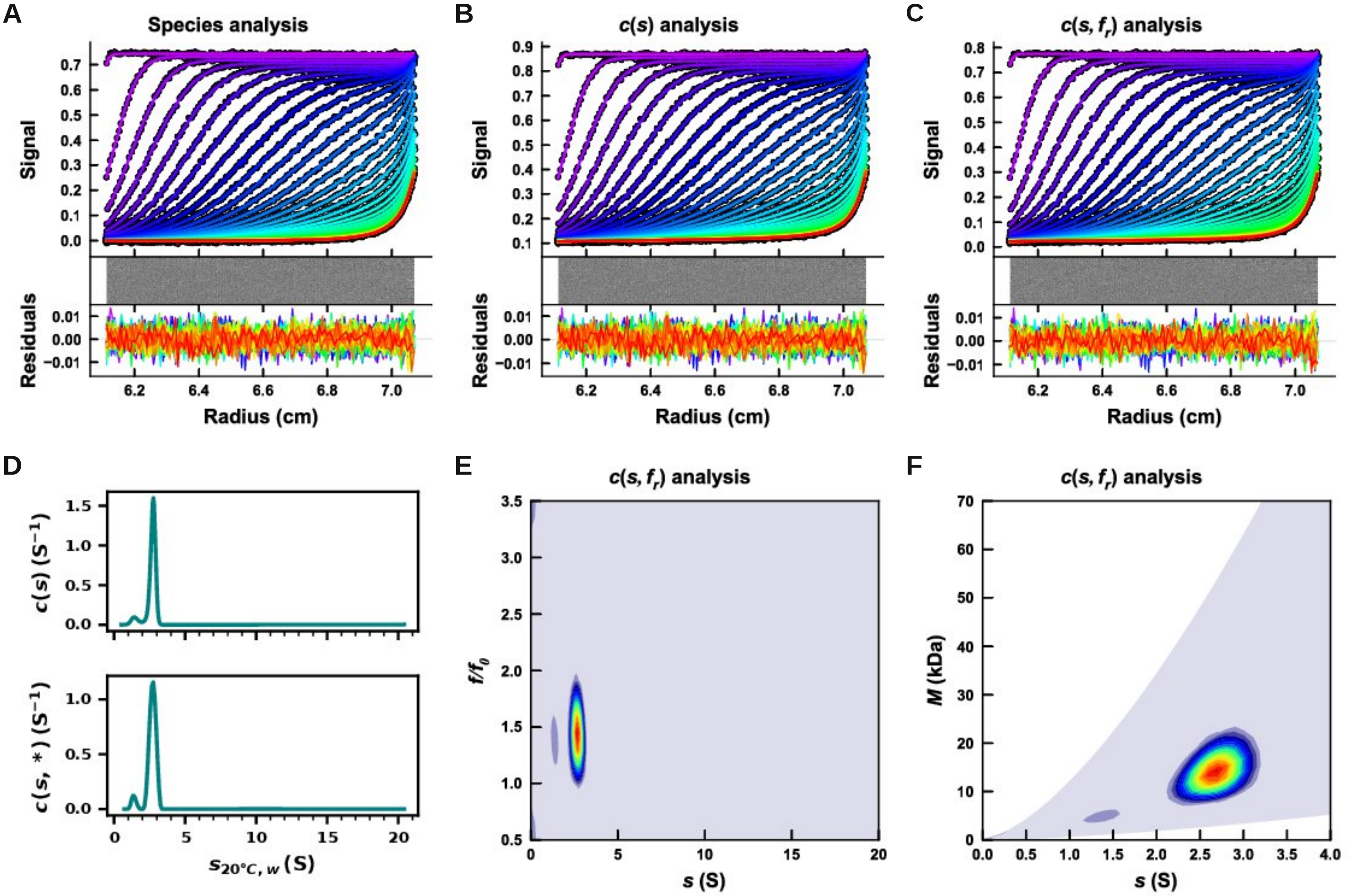

Fig. S8J: Data, fit and residuals[5] of **(A)** species analysis[2] with 2 species, **(B)** *c(s)* analysis[4] and **(C)** *c(s, f_r)* analysis[3] in Sedfit. **(D)** The top panel shows the *c(s)* distribution and the bottom panel the *c(s, *)* distribution. *s* values were corrected to standard conditions. **(E)** Plot of frictional ratio f_r versus uncorrected sedimentation coefficient *s* as obtained from *c(s, f_r)* analysis. **(F)** Plot of molecular mass *M* versus uncorrected sedimentation coefficient *s* as obtained from *c(s, f_r)* analysis.

Species	Parameter	Species analysis		*c*(*s*) analysis	*c*(*s*, f_r) analysis
	**Fit r.m.s.d.**	0.003719		0.003692	0.003695
		**Best fit value**	**95% confidence interval**		
**Monomer**	$s_{20°C,w}$ **(S)**	1.59	1.40 - 1.79	1.46	1.39
	M **(Da)**	5120	4580 - 5700		4970
	f_r				1.42
	**Fraction**	0.101	0.081 - 0.131	0.085	0.067
**Dimer**	$s_{20°C,w}$ **(S)**	2.74	2.71- 2.77	2.77	2.72
	M **(Da)**	13270	12740 - 13880		13880
	f_r				1.43
	**Fraction**	0.899	0.869 - 0.919	0.915	0.933

Supplementary Table S5J: SV analysis of hTR 1-20 DNA c2 at a concentration 0.050 mg/ml (8.0 µM) in 20 mM HEPES, pH 7.5, 100 mM KCl using a partial specific volume $\bar{\nu}$ of 0.541 cm^3/g, a solvent density ρ of 1.00450 g/cm^3 and solvent viscosity η of 0.01015 P. All sedimentation coefficients were corrected to standard conditions ($s_{20°C,w}$). Other parameters shown are the molecular mass *M*, frictional ratio f_r and the relative fractions of the two species.

Experiment # 1 @ 0.010 mg/ml (1.6 µM) hTR 1-20 DNA c2

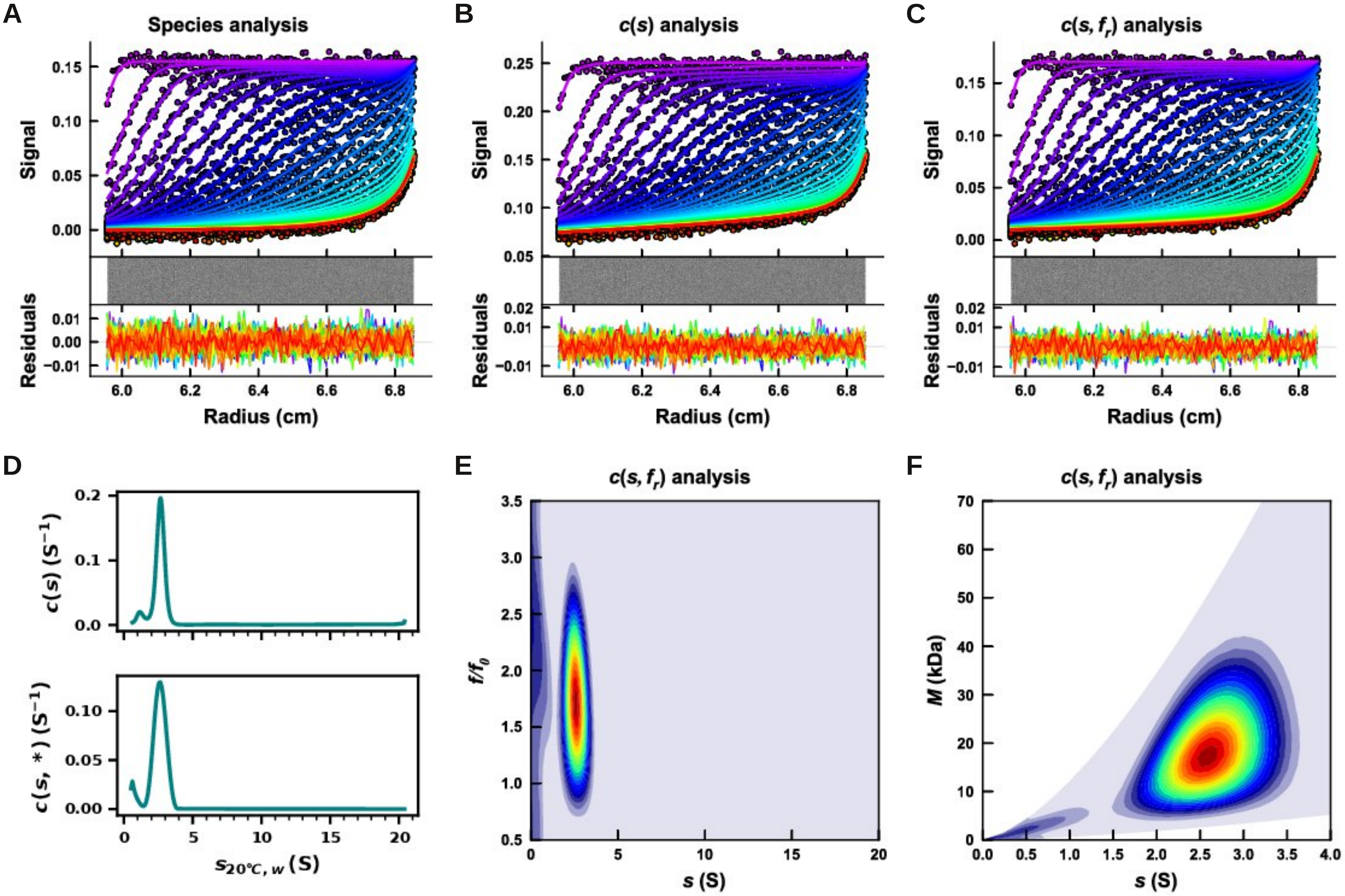

Fig. S8K: Data, fit and residuals[5] of **(A)** species analysis[2] with 2 species, **(B)** *c(s)* analysis[4] and **(C)** *c(s, f_r)* analysis[3] in Sedfit. **(D)** The top panel shows the *c(s)* distribution and the bottom panel the *c(s, *)* distribution. *s* values were corrected to standard conditions. **(E)** Plot of frictional ratio f_r versus uncorrected sedimentation coefficient *s* as obtained from *c(s, f_r)* analysis. **(F)** Plot of molecular mass *M* versus uncorrected sedimentation coefficient *s* as obtained from *c(s, f_r)* analysis.

Species	Parameter	Species analysis		*c*(*s*) analysis	*c*(*s*, f_r) analysis
	**Fit r.m.s.d.**	0.003641		0.003638	0.003578
		**Best fit value**	**95% confidence interval**		
**Monomer**	$s_{20°C,w}$ **(S)**	1.31	0.76 - 1.93	1.19	n/a
	M **(Da)**	3400	2100 - 4960		n/a
	f_r				n/a
	**Fraction**	0.122	0.088 - 0.211	0.092	n/a
**Dimer**	$s_{20°C,w}$ **(S)**	2.67	2.58 - 2.76	2.66	2.62
	M **(Da)**	13640	11540 - 16540		17370
	f_r				1.73
	**Fraction**	0.878	0.789 - 0.912	0.908	n/a

Supplementary Table S5K: SV analysis of hTR 1-20 DNA c2 at a concentration 0.010 mg/ml (1.6 µM) in 20 mM HEPES, pH 7.5, 100 mM KCl using a partial specific volume $\bar{\nu}$ of 0.541 cm^3/g, a solvent density ρ of 1.00450 g/cm^3 and solvent viscosity η of 0.01015 P. All sedimentation coefficients were corrected to standard conditions ($s_{20°C,w}$). Other parameters shown are the molecular mass *M*, frictional ratio f_r and the relative fractions of the two species.

Experiment # 2 @ 0.010 mg/ml (1.6 µM) hTR 1-20 DNA c2

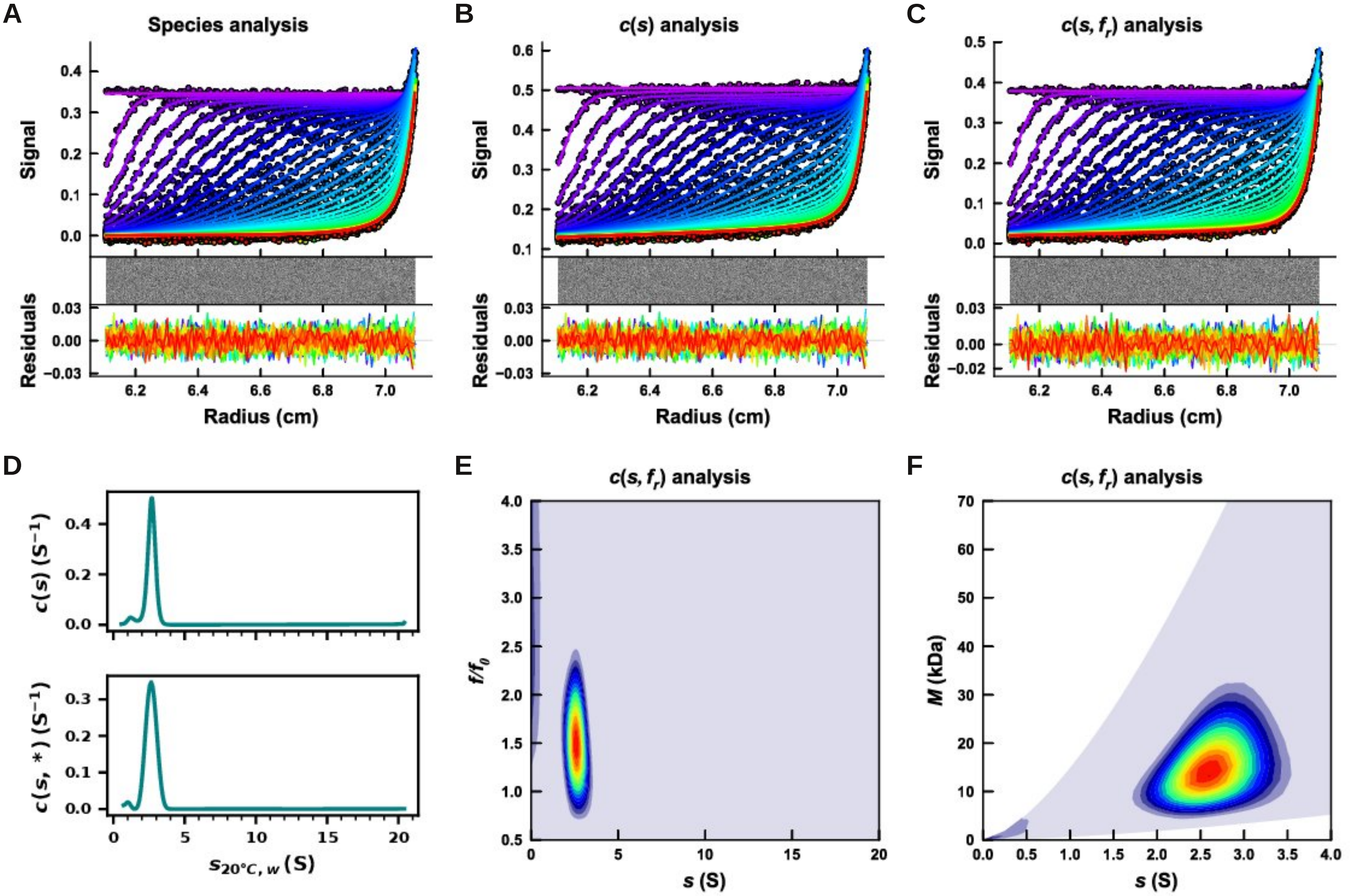

Fig. S8L: Data, fit and residuals[5] of **(A)** species analysis[2] with 2 species, **(B)** *c(s)* analysis[4] and **(C)** *c(s, f_r)* analysis[3] in Sedfit. **(D)** The top panel shows the *c(s)* distribution and the bottom panel the *c(s, *)* distribution. *s* values were corrected to standard conditions. **(E)** Plot of frictional ratio f_r versus uncorrected sedimentation coefficient *s* as obtained from *c(s, f_r)* analysis. **(F)** Plot of molecular mass *M* versus uncorrected sedimentation coefficient *s* as obtained from *c(s, f_r)* analysis.

Species	Parameter	Species analysis		*c*(*s*) analysis	*c*(*s*, f_r) analysis
	**Fit r.m.s.d.**	0.007096		0.007093	0.007081
		**Best fit value**	**95% confidence interval**		
**Monomer**	$s_{20°C,w}$ **(S)**	1.34	0.75 - 1.94	1.24	n/a
	M **(Da)**	3830	2270 - 5850		n/a
	f_r				n/a
	**Fraction**	0.086	0.058 - 0.169	0.053	0.038
**Dimer**	$s_{20°C,w}$ **(S)**	2.68	2.61- 2.77	2.69	2.66
	M **(Da)**	12330	10830 - 14530		14480
	f_r				1.51
	**Fraction**	0.914	0.831 - 0.942	0.947	0.962

Supplementary Table S5L: SV analysis of hTR 1-20 DNA c2 at a concentration 0.010 mg/ml (1.6 µM) in 20 mM HEPES, pH 7.5, 100 mM KCl using a partial specific volume $\bar{\nu}$ of 0.541 cm^3/g, a solvent density ρ of 1.00450 g/cm^3 and solvent viscosity η of 0.01015 P. All sedimentation coefficients were corrected to standard conditions ($s_{20°C,w}$). Other parameters shown are the molecular mass *M*, frictional ratio f_r and the relative fractions of the two species.

0.005 mg/ml (0.8 µM) hTR 1-20 DNA c2

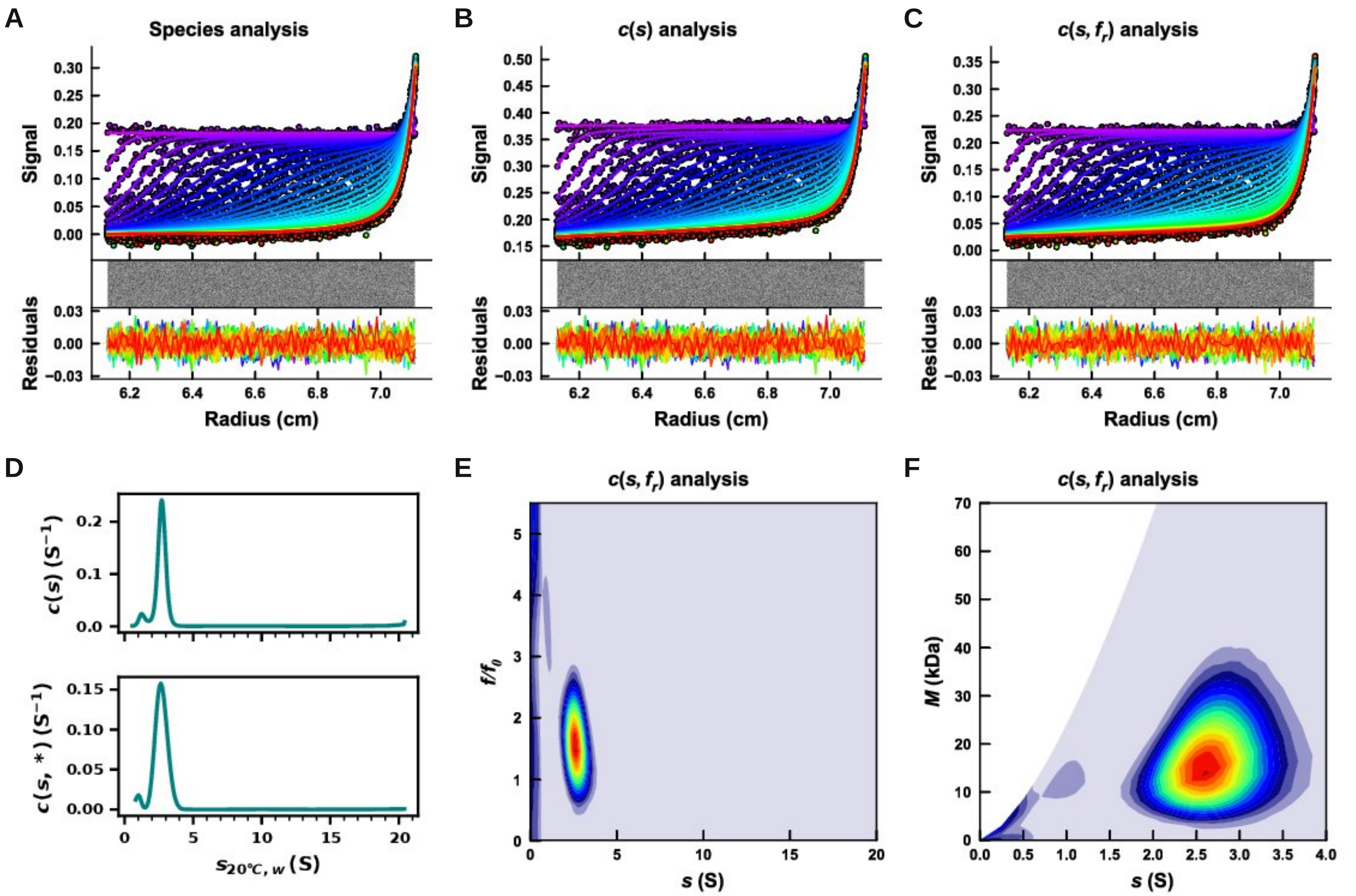

Fig. S8M: Data, fit and residuals[5] of **(A)** species analysis[2] with 2 species, **(B)** *c(s)* analysis[4] and **(C)** *c(s, f_r)* analysis[3] in Sedfit. **(D)** The top panel shows the *c(s)* distribution and the bottom panel the *c(s, *)* distribution. *s* values were corrected to standard conditions. **(E)** Plot of frictional ratio f_r versus uncorrected sedimentation coefficient *s* as obtained from *c(s, f_r)* analysis. **(F)** Plot of molecular mass *M* versus uncorrected sedimentation coefficient *s* as obtained from *c(s, f_r)* analysis.

Species	Parameter	Species analysis		*c*(*s*) analysis	*c*(*s*, f_r) analysis
	**Fit r.m.s.d.**	0.007210		0.007208	0.007198
		**Best fit value**	**95% confidence interval**		
**Monomer**	$s_{20°C,w}$ **(S)**	1.25	0.56 - 2.00	1.27	1.00
	***M* (Da)**	2910	1370 - 5020		12200
	f_r				3.60
	**Fraction**	0.113	0.080 - 0.191	0.074	0.048
**Dimer**	$s_{20°C,w}$ **(S)**	2.72	2.58 - 2.88	2.72	2.70
	***M* (Da)**	12160	10000- 15730		15800
	f_r				1.58
	**Fraction**	0.887	0.809 - 0.920	0.926	0.952

Supplementary Table S5M: SV analysis of hTR 1-20 DNA c2 at a concentration 0.005 mg/ml (0.8 µM) in 20 mM HEPES, pH 7.5, 100 mM KCl using a partial specific volume $\bar{\nu}$ of 0.541 cm^3/g, a solvent density ρ of 1.00450 g/cm^3 and solvent viscosity η of 0.01015 P. All sedimentation coefficients were corrected to standard conditions ($s_{20°C,w}$). Other parameters shown are the molecular mass *M*, frictional ratio f_r and the relative fractions of the two species.

References

1 Meier, M. *et al.* Binding of G-quadruplexes to the N-terminal recognition domain of the RNA helicase associated with AU-rich element (RHAU). *J Biol Chem* **288**, 35014-35027, doi:10.1074/jbc.M113.512970 (2013).

2 Schuck, P. Sedimentation analysis of noninteracting and self-associating solutes using numerical solutions to the Lamm equation. *Biophys J* **75**, 1503-1512, doi:10.1016/S0006-3495(98)74069-X (1998).

3 Brown, P. H. & Schuck, P. Macromolecular size-and-shape distributions by sedimentation velocity analytical ultracentrifugation. *Biophys J* **90**, 4651-4661, doi:10.1529/biophysj.106.081372 (2006).

4 Schuck, P. Size-distribution analysis of macromolecules by sedimentation velocity ultracentrifugation and lamm equation modeling. *Biophys J* **78**, 1606-1619, doi:10.1016/S0006-3495(00)76713-0 (2000).

5 Brautigam, C. A. Calculations and Publication-Quality Illustrations for Analytical Ultracentrifugation Data. *Methods Enzymol* **562**, 109-133, doi:10.1016/bs.mie.2015.05.001 (2015).

6 Lu, X. J., Bussemaker, H. J. & Olson, W. K. DSSR: an integrated software tool for dissecting the spatial structure of RNA. *Nucleic Acids Res* **43**, e142, doi:10.1093/nar/gkv716 (2015).

7 Lu, X. J. & Olson, W. K. 3DNA: a software package for the analysis, rebuilding and visualization of three-dimensional nucleic acid structures. *Nucleic Acids Res* **31**, 5108-5121, doi:10.1093/nar/gkg680 (2003).

8 Kantardjieff, K. A. & Rupp, B. Matthews coefficient probabilities: Improved estimates for unit cell contents of proteins, DNA, and protein-nucleic acid complex crystals. *Protein Sci* **12**, 1865-1871, doi:10.1110/ps.0350503 (2003).

9 Weichenberger, C. X. & Rupp, B. Ten years of probabilistic estimates of biocrystal solvent content: new insights via nonparametric kernel density estimate. *Acta Crystallogr D Biol Crystallogr* **70**, 1579-1588, doi:10.1107/S1399004714005550 (2014).

10 Svergun, D. I. Restoring low resolution structure of biological macromolecules from solution scattering using simulated annealing (vol 76, pg 2879, 1999). *Biophysical Journal* **77**, 2896-2896, doi:10.1016/S0006-3495(99)77443-6 (1999).

11 Franke, D. & Svergun, D. I. DAMMIF, a program for rapid ab-initio shape determination in small-angle scattering. *J Appl Crystallogr* **42**, 342-346, doi:10.1107/S0021889809000338 (2009).

12 Svergun, D. I. Determination of the Regularization Parameter in Indirect-Transform Methods Using Perceptual Criteria. *Journal of Applied Crystallography* **25**, 495-503, doi:10.1107/S0021889892001663 (1992).

13 Valentini, E., Kikhney, A. G., Previtali, G., Jeffries, C. M. & Svergun, D. I. SASBDB, a repository for biological small-angle scattering data. *Nucleic Acids Res* **43**, D357-363, doi:10.1093/nar/gku1047 (2015).

14 Garcia De La Torre, J., Huertas, M. L. & Carrasco, B. Calculation of hydrodynamic properties of globular proteins from their atomic-level structure. *Biophys J* **78**, 719-730, doi:10.1016/S0006-3495(00)76630-6 (2000).

15 Garcia de la Torre, J., Harding, S. E. & Carrasco, B. Calculation of NMR relaxation, covolume, and scattering-related properties of bead models using the SOLPRO computer program. *Eur Biophys J* **28**, 119-132, doi:10.1007/s002490050191 (1999).

16 Garcia de la Torre, J. Hydration from hydrodynamics. General considerations and applications of bead modelling to globular proteins. *Biophys Chem* **93**, 159-170, doi:10.1016/S0301-4622(01)00218-6 (2001).

17 Phillips, K., Dauter, Z., Murchie, A. I., Lilley, D. M. & Luisi, B. The crystal structure of a parallel-stranded guanine tetraplex at 0.95 A resolution. *J Mol Biol* **273**, 171-182, doi:10.1006/jmbi.1997.1292 (1997).

18 Parkinson, G. N., Cuenca, F. & Neidle, S. Topology conservation and loop flexibility in quadruplex-drug recognition: crystal structures of inter- and intramolecular telomeric DNA quadruplex-drug complexes. *J Mol Biol* **381**, 1145-1156, doi:10.1016/j.jmb.2008.06.022 (2008).

19 Campbell, N. H. *et al.* Molecular basis of structure-activity relationships between salphen metal complexes and human telomeric DNA quadruplexes. *J Med Chem* **55**, 209-222, doi:10.1021/jm201140v (2012).

20 Do, N. Q., Lim, K. W., Teo, M. H., Heddi, B. & Phan, A. T. Stacking of G-quadruplexes: NMR structure of a G-rich oligonucleotide with potential anti-HIV and anticancer activity. *Nucleic Acids Res* **39**, 9448-9457, doi:10.1093/nar/gkr539 (2011).

21 Trajkovski, M., da Silva, M. W. & Plavec, J. Unique structural features of interconverting monomeric and dimeric G-quadruplexes adopted by a sequence from the intron of the N-myc gene. *J Am Chem Soc* **134**, 4132-4141, doi:10.1021/ja208483v (2012).

22 The PyMOL Molecular Graphics System v. 2.0 (Schrödinger, LLC, 2017).
